# Biases in bulk: DNA metabarcoding of marine communities and the methodology involved

**DOI:** 10.1111/mec.15592

**Published:** 2020-08-29

**Authors:** Luna M. van der Loos, Reindert Nijland

**Affiliations:** ^1^ Marine Animal Ecology Group Wageningen University Wageningen The Netherlands; ^2^ Present address: Department of Biology Phycology Research Group Ghent University Ghent Belgium

**Keywords:** biodiversity, DNA metabarcoding, marine monitoring, technical biases

## Abstract

With the growing anthropogenic pressure on marine ecosystems, the need for efficient monitoring of biodiversity grows stronger. DNA metabarcoding of bulk samples is increasingly being implemented in ecosystem assessments and is more cost‐efficient and less time‐consuming than monitoring based on morphology. However, before raw sequences are obtained from bulk samples, a profound number of methodological choices must be made. Here, we critically review the recent methods used for metabarcoding of marine bulk samples (including benthic, plankton and diet samples) and indicate how potential biases can be introduced throughout sampling, preprocessing, DNA extraction, marker and primer selection, PCR amplification and sequencing. From a total of 64 studies evaluated, our recommendations for best practices include to (a) consider DESS as a fixative instead of ethanol, (b) use the DNeasy PowerSoil kit for any samples containing traces of sediment, (c) not limit the marker selection to COI only, but preferably include multiple markers for higher taxonomic resolution, (d) avoid touchdown PCR profiles, (e) use a fixed annealing temperature for each primer pair when comparing across studies or institutes, (f) use a minimum of three PCR replicates, and (g) include both negative and positive controls. Although the implementation of DNA metabarcoding still faces several technical complexities, we foresee wide‐ranging advances in the near future, including improved bioinformatics for taxonomic assignment, sequencing of longer fragments and the use of whole‐genome information. Despite the bulk of biases involved in metabarcoding of bulk samples, if appropriate controls are included along the data generation process, it is clear that DNA metabarcoding provides a valuable tool in ecosystem assessments.

## 1 INTRODUCTION

Marine ecosystem health is inextricably linked to biodiversity (Goodwin et al., 2017; Porter & Hajibabaei, 2018b). Monitoring marine biodiversity in an accurate and cost‐effective fashion is essential in order to assess ecosystem quality (Aylagas, Borja, & Rodríguez‐Ezpeleta, 2014). This becomes increasingly important in the face of anthropogenic pressure and management of human activities (Leray & Knowlton, 2016). However, traditional biodiversity assessments require identifying each specimen in a sample using morphological characteristics and taxonomic keys. This labour‐intensive and time‐consuming work needs experienced taxonomists and is therefore an expensive method (Leray & Knowlton, 2016). DNA metabarcoding, which is the simultaneous identification of many taxa within the same sample based on high‐throughput sequencing of the pooled genomic DNA, provides a time‐ and cost‐effective alternative (Aylagas, Borja, Muxika, & Rodríguez‐Ezpeleta, 2018).

Genomic DNA can be extracted from either bulk samples (e.g., organisms isolated from sediment collected with sediment cores or grabs, or organisms collected using plankton trawls) or from environmental samples (e.g., water or sediment samples; Creer et al., 2016). The DNA in bulk samples, often referred to as community DNA, is isolated from the tissue of the specimens present in the sample. Environmental DNA (eDNA), on the other hand, concerns DNA obtained directly from environmental samples without the presence of biological source material (Deiner et al., 2017). Although metabarcoding often refers to both type of samples, they differ widely in the quantity and quality of the DNA and the applications. Bulk samples contain whole specimens and thus whole genomic DNA of relatively high quality, whereas environmental samples contain very little relevant DNA and which also is mostly degraded (Deiner et al., 2017). As a consequence, metabarcoding of bulk samples (DNA metabarcoding) is mainly used when samples are relatively easy to obtain, whereas metabarcoding of environmental samples (eDNA metabarcoding) is more useful when targeting organisms that are difficult to sample (e.g., fish). Samples concerning dietary analyses (i.e., using faeces and/or stomach content to study the diet of an organism) are in the grey area between bulk and environmental samples, as they do contain biological source material, but the DNA is often degraded and therefore of lower quality (Creer et al., 2016; Pompanon et al., 2012). Here, metabarcoding is especially valuable, as morphological characteristics are often hard to identify in the partially digested organisms in stomach or faeces content.

DNA metabarcoding of bulk samples has been used in assessing marine biodiversity for over a decade. Studies have included benthic invertebrates in sediment (e.g., Chariton, Court, Hartley, Colloff, & Hardy, 2010; Fonseca et al., 2010), plankton communities (e.g., Nanjappa, Audic, Romac, Kooistra, & Zingone, 2014; Zaiko, Samuiloviene, Ardura, & Garcia‐Vazquez, 2015), hard substrate samples (e.g., Leray & Knowlton, 2015; Pearman, Anlauf, Irigoien, & Carvalho, 2016) and dietary samples (e.g., Albaina, Aguirre, Abad, Santos, & Estonba, 2016; Yoon et al., 2017). Since then, DNA metabarcoding has been increasingly used worldwide in marine samples, as is demonstrated by projects such as the Genetic tools for Ecosystem health Assessment in the North Sea region (GEANS) and the Global ARMS Program with Autonomous Reef Monitoring Structures.

**FIGURE 1:**
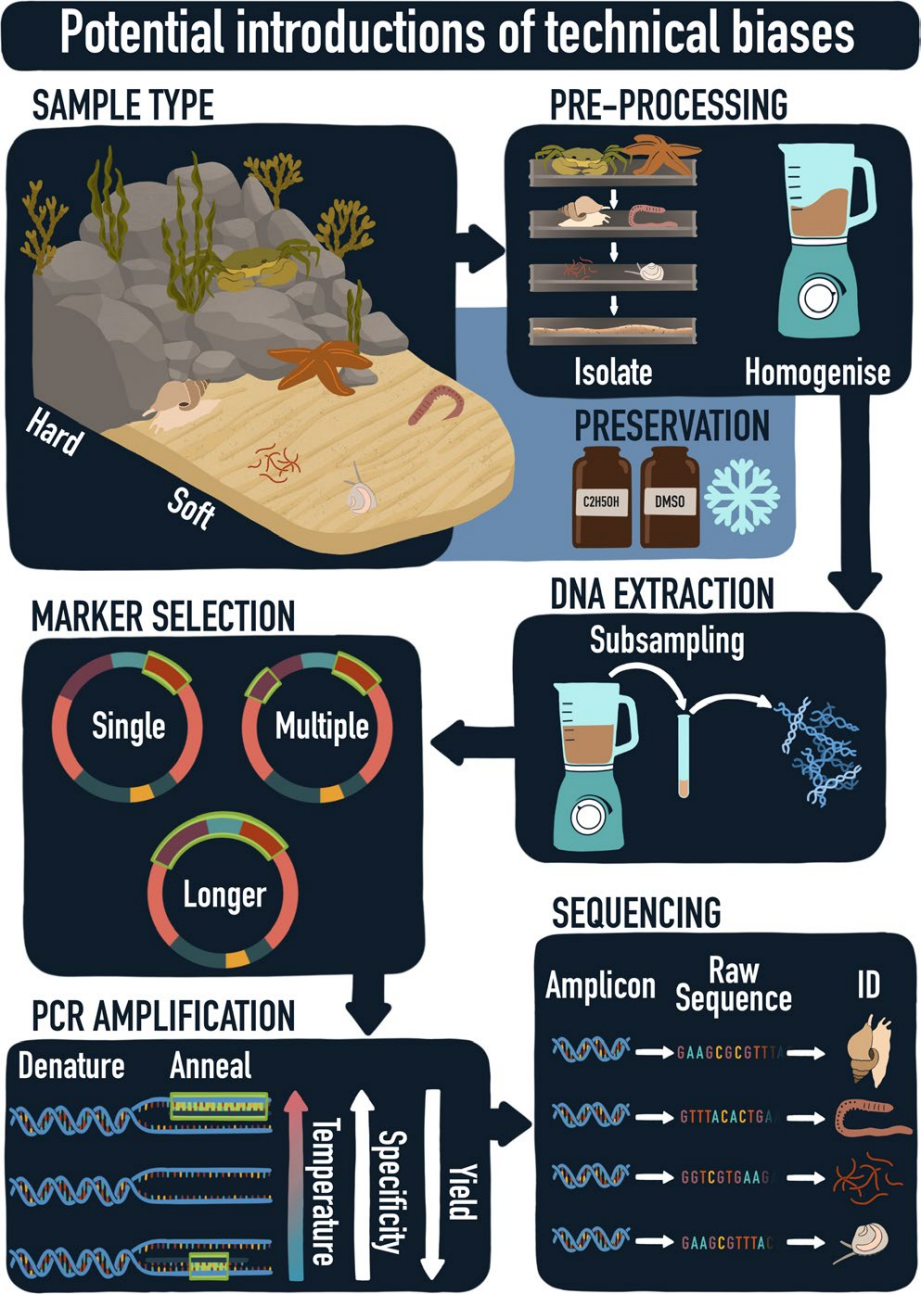
Schematic overview of the process from bulk sample to raw sequences and taxonomic assignments. Technical biases can potentially be introduced during sampling, preprocessing, DNA extraction, marker selection, PCR amplification and sequencing. (Graphical design by Aline Joustra)

**FIGURE 2:**
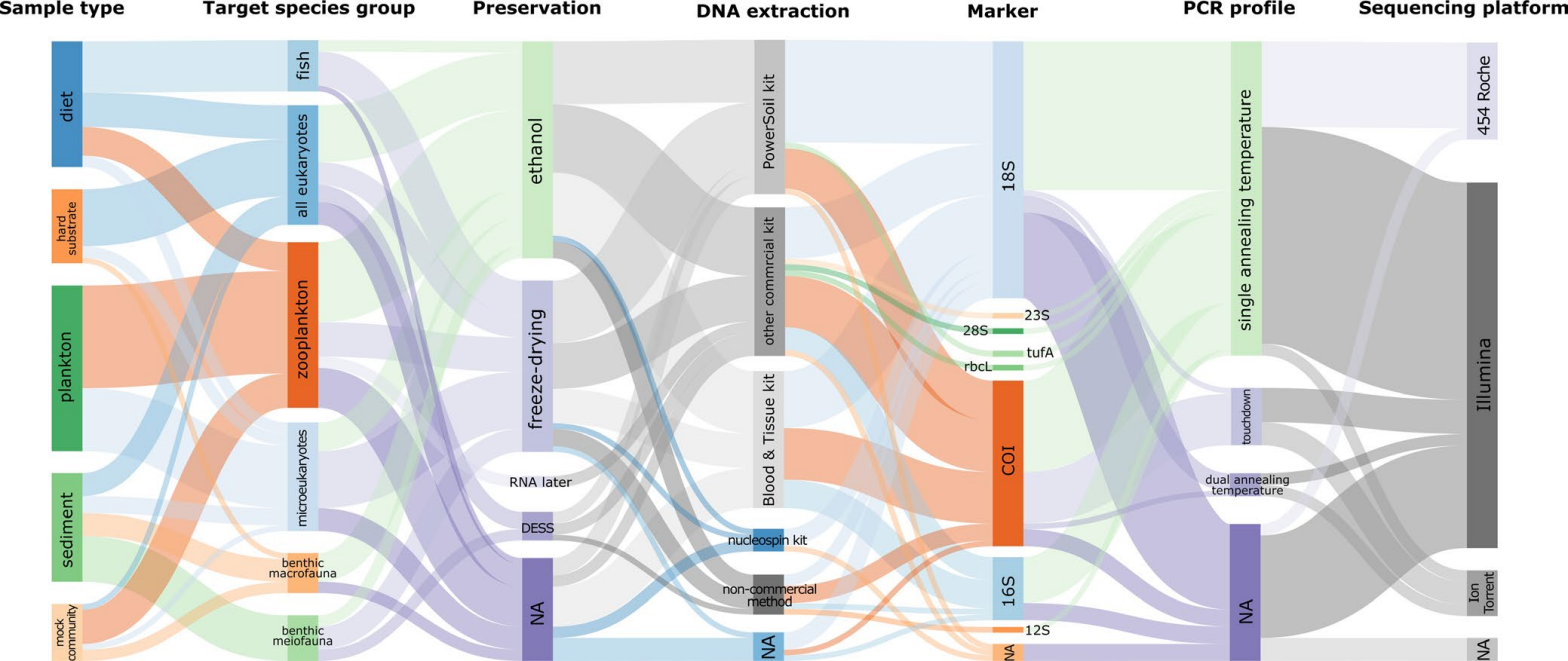
Relative frequencies of methods used during seven methodological stages in DNA metabarcoding of bulk samples from 64 studies published between 2010 and 2019, including sample type, target species group, preservation, DNA extraction, marker, PCR profile and sequencing platform. The thickness of the lines corresponds to the number of papers using this method. “NA” represents studies where the corresponding stage/method was not reported or was not needed. The diagram was created with sankeymatic (http://sankeymatic.com/build/)

The increased use of DNA metabarcoding allows for comparisons across studies and within time‐series but simultaneously calls for the need of harmonized and standardized protocols and the implementation of DNA‐based methods in standard routine monitoring programmes. Many methodological steps separate the moment of sampling from obtaining raw sequences and taxonomic assignments from these bulk samples. Starting with the sample method itself, many choices have to be made (e.g., choices of target species group, sampling period, sample type, and (sub)sample size). This continues throughout the process with different preprocessing options (e.g., fixation method, separating the organic from the inorganic compound, size fractioning), DNA extraction methods, DNA marker regions and primers, PCR (polymerase chain reaction) amplification profiles, and finally sequencing (summarized in Figure 1). A wide array of methods are currently in use, resulting in an overwhelming number of methodological options to choose from (Figure 2). Deciding which method or choice is most suitable during each step is often highly complex, as the complete experimental design and sample types will have to be considered. Technical biases can be introduced with every step in this process and are likely to have a large impact on the obtained results. Harmonization and standardization of methods are therefore essential for DNA metabarcoding to become a standard for biodiversity monitoring. Although standardization of methods will not remove these biases, it will increase the degree to which the results of different studies can be compared. In addition, harmonization of methods is not limited to specifying the standard operating procedures, but also extends to what practices should be avoided. Understanding when and how technical biases can be introduced is a first step towards minimizing the impact of these technical biases and reaching harmonized protocols.

In this review, we focus on the methodology behind DNA metabarcoding of marine bulk samples, including benthic communities of sediment and hard substrate, plankton samples and dietary samples. Although metabarcoding of eDNA from environmental samples has greatly increased in popularity, bulk samples of community DNA yield better results for benthic communities as of now. The methodology of eDNA has received considerable attention—e.g., Creer et al. (2016) and Deiner et al. (2017)—but the differences in quality and quantity of bulk DNA and eDNA will probably result in different best practice protocols for bulk DNA metabarcoding (Klunder et al., 2019). A concise review focusing on the technical biases and bottlenecks associated with DNA metabarcoding of bulk samples has hitherto been lacking. The aim of this review is three‐fold: (a) to provide an overview of currently employed methods and the potential biases involved with bulk DNA metabarcoding, based on a comprehensive literature review and a survey, (b) to offer recommendations for best practice protocols and standard procedures, and (c) to critically assess the current bottlenecks and expected future improvements in relation to the implementation of bulk DNA metabarcoding in existing monitoring programmes.

## 2 METHODS

**FIGURE 3:**
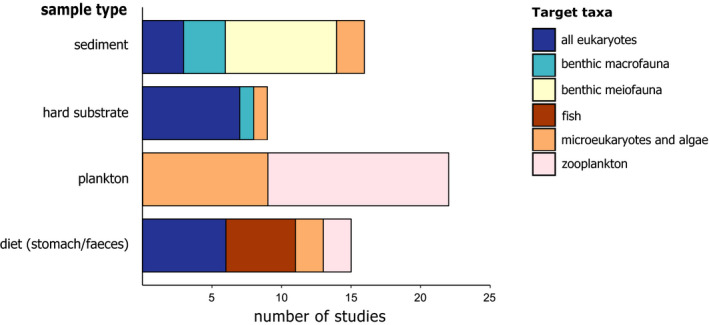
Taxa targeted in bulk DNA metabarcoding studies with different sample types

**FIGURE 4:**
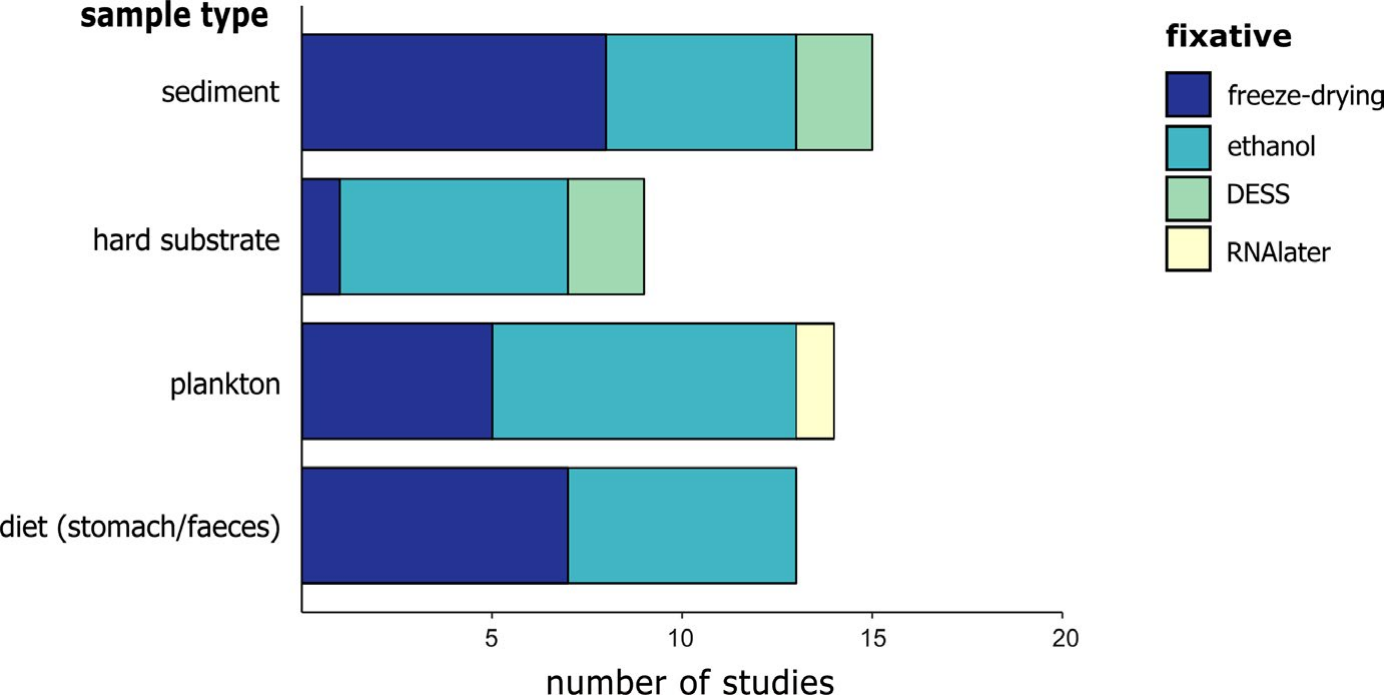
Preservation method used in bulk metabarcoding studies with different sample types

**FIGURE 5:**
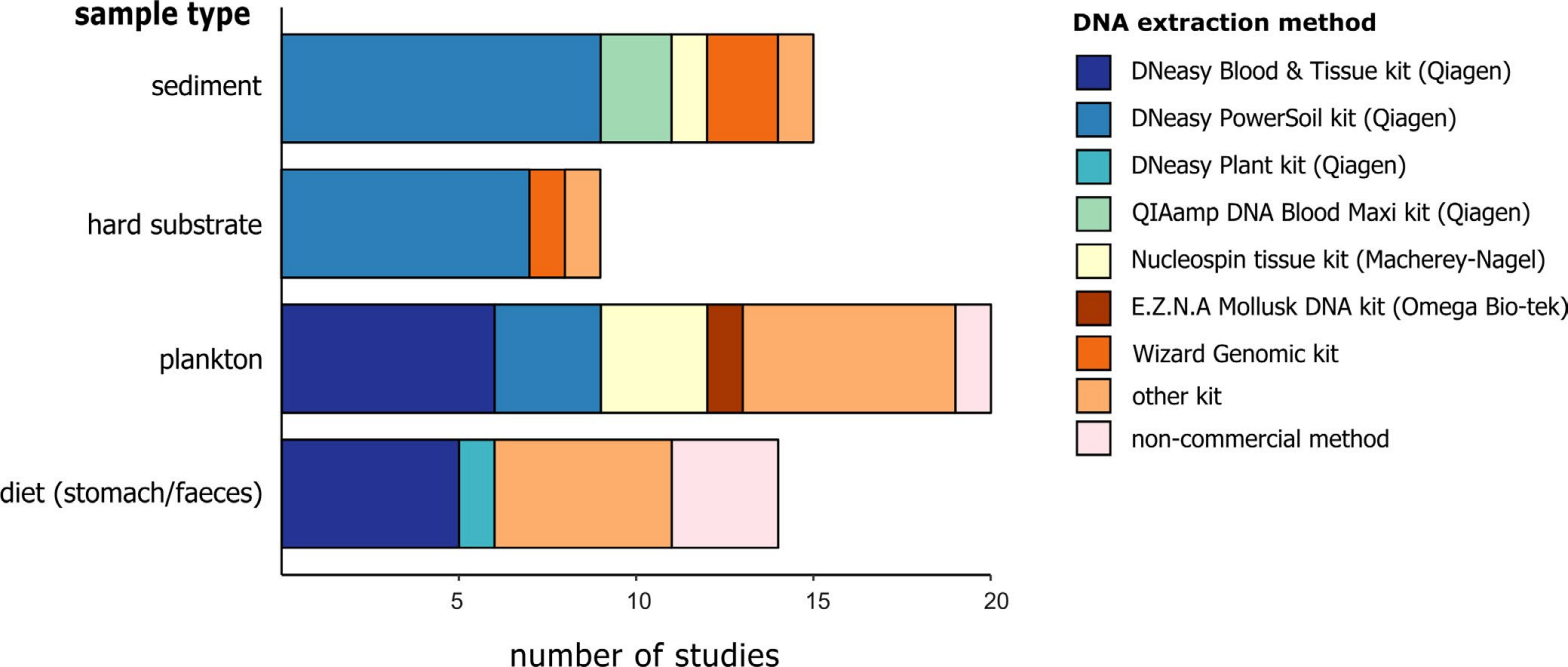
DNA extraction methods used in bulk metabarcoding studies with different sample types

**FIGURE 6:**
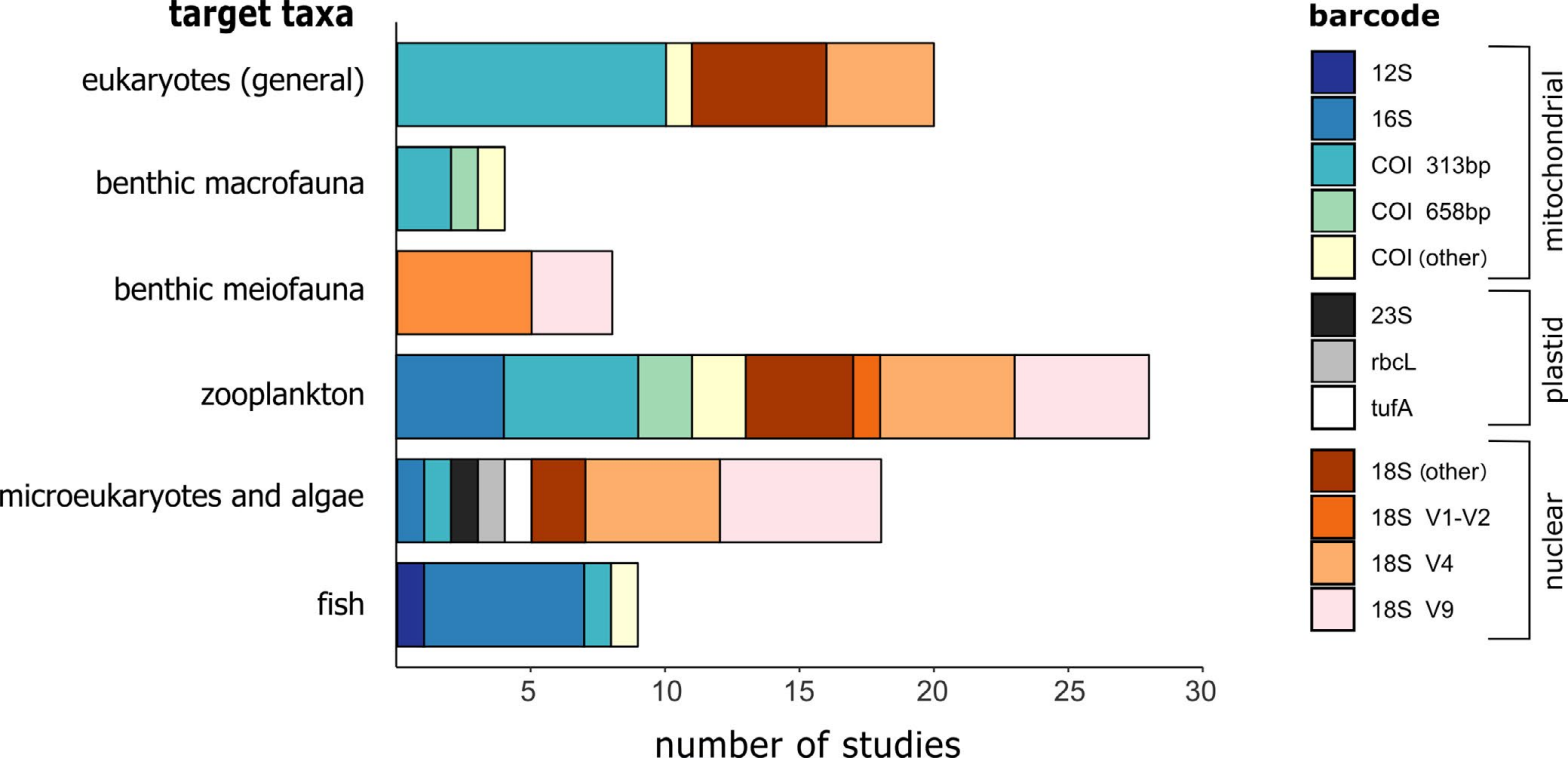
Different available markers used in bulk metabarcoding studies when targeting different marine groups

To synthesize and do a “stock take” of recent approaches and methods used for DNA metabarcoding of bulk samples, we conducted a systematic literature survey. We searched the Web of Science database in April 2019 using the key words “DNA metabarcoding,” “marine,” “benthos,” “fauna,” “biodiversity,” “meiofauna,” “macrofauna,” “sediment,” “diet” and “hard substrate” in various combinations of two or three keywords, resulting in 518 returned records. Duplicate papers were removed as well as those that were not primary research articles, not published in peer‐reviewed journals and not written in English. Following initial filtering, a total of 133 studies were considered for future reading, with data extracted only from papers that concerned (a) eukaryotic organisms, (b) organisms sampled in a marine environment and (c) DNA metabarcoding of bulk samples. After excluding all studies that did not meet these criteria, 64 studies published between 2010 and 2019 were analysed. For each study, we extracted information about the sample type (plankton, soft sediment, hard substrate or stomach content/faeces), the target species groups, sample preprocessing, the preservation method, the DNA extraction method, the selected markers (target DNA region and primers), the PCR thermal profile, the number of technical PCR replicates and the sequencing platform used (see Materials S1). The results were visualized in two types of graphs: a Sankey diagram and bar graphs. The Sankey diagram (Figure 2) provides an overview of the methods used by the 64 studies included in the literature review using flows of which the size is proportional to the relative frequencies. This diagram was created using sankeymatic (http://sankeymatic.com/build/). The bar graphs (Figures 3, 4, 5, 6) provide more detailed information showing the number of studies using a specific method in relation to the sample type and target taxa. Note that the number of studies in the bar graphs does not always equal 64, as not all methods applied to every single study (e.g., preservation method of bulk samples did not apply to studies using an artificially assembled mock community). All bar graphs have been created using R 3.6.1 (R Core Team, 2019) with the package ggplot2 (Wickham, 2016).

The publications included in this review span nearly a decade (2010–2019). During this time, metabarcoding techniques have improved significantly and methods used 10 years ago are likely to be outdated by now. To identify the most current methods used by the metabarcoding community, nine European institutes were asked to fill in a questionnaire related to the processing and molecular methods of marine samples. These institutes currently collaborate in the EU Interreg North Sea region project GEANS. The responses (*n* = 9) to the questionnaire provided insights supplementing the results from the literature survey, and identified the current commonly used methods, as well as a few key points for improvements and recommendations. Although all the graphs in this paper only present the results of the literature review, we combined the information from both the literature survey and the questionnaire in designing the recommendations given in Section 4. Details of the questionnaire and a summary of the answers are given in Materials S2 (Figures S1–S6 and Table S1).

## 3 RESULTS AND DISCUSSION

### 3.1 Sample type and target species

DNA metabarcoding of bulk samples is used to reveal the biodiversity associated with a wide variety of habitats and sample types in the marine environment. The main sample types include soft sediment (27% of the publications included in this study), hard substrate (19%), diet (i.e., faeces or stomach contents; 24% of the studies) and plankton samples (30%). DNA metabarcoding of bulk samples is especially useful for the latter two sample types, as the organisms (and their remnants) in these samples are often hard to identify. Soft sediment is relatively easy to sample using cores or grabs (Aylagas, Mendibil, Borja, & Rodríguez‐Ezpeleta, 2016). Sampling hard substrate is more complex due to the three‐dimensional environment. Classic methods include scraping quadrats or using settlement plates. Since 2015, Autonomous Reef Monitoring Structures (ARMS) have been deployed for use in DNA metabarcoding (Leray & Knowlton, 2015). These structures, consisting of nine stacked PVC plates, are similar to settlement plates, but the three‐dimensional character of the structure also attracts motile organisms. This additionally allows for sampling of organisms that hide in cracks and crevices and are otherwise hard to count during visual surveys.

The targeted species group is another important concern in the study design, as this also affects the methods used in processing the samples, and the primer set and marker selection later on. Traditionally, benthic meiofauna and macrofauna are used as bio‐indicators in morphology‐based studies to assess environmental status of many soft sediment‐dominated ecosystems (Dauvin, Bellan, & Bellan‐Santini, 2010; Moreno et al., 2008). Hence, the focus of DNA metabarcoding fauna from soft sediment samples is predominantly on invertebrates (Figure 3). Hard substrate samples typically target eukaryotes in general (i.e., they are not restricted to invertebrates), but also include protists such as Rhodophyta. Plankton is mainly targeted in the context of early detection of nonindigenous species. Studies analysing diet composition target a range of different taxa, depending on the feeding habit of the study organism (e.g., herbivorous, piscivorous, planktivorous). DNA metabarcoding of bulk samples aiming for fish species is applied only in diet‐based studies of their predatory species (Figure 3), as metabarcoding of eDNA in water samples is generally regarded as being more suitable for monitoring fish diversity due to the high sampling effort associated with traditional fish samples (Evans et al., 2017).

### 3.2 Sample preservation

The quality of DNA in a sample can degrade quickly, especially when working in high temperatures (Dawson, Raskoff, & Jacobs, 1998). Samples are therefore required to be either immediately preserved after collection in the field, or immediately preprocessed on‐site and subsequently fixed. There are several options available for the preservation of samples, including ethanol, freezing or freeze‐drying, RNAlater and DESS (salt saturated DMSO buffers containing EDTA). Our literature survey showed that 96%–100% ethanol is the most widely used method (50% of analysed studies), especially for plankton and hard substrate (Figure 4). In sediment and diet samples (stomach/faeces content), freezing is also widely used. DESS is only used in 9% of all studies, predominantly with meiofauna and nematode research. The reason DESS is overall less popular than ethanol can partly be attributed to habits. Formalin has a long tradition in preservation of specimens for morphological research but was replaced by ethanol for molecular studies after it became clear that formalin was unsuitable for molecular analyses. In addition, ethanol is very accessible as it is available in every laboratory. However, ethanol is less adequate in preserving the morphological characters of specimens and is furthermore a restricted chemical in relation to shipping. DMSO, on the other hand, is not a restricted chemical (Williams, 2007) and samples conserved in DESS are thus more easily transported and shipped than samples preserved with ethanol. In search for a more ideal method than formalin and ethanol, Yoder et al. (2006) showed that DESS ensured adequate preservation of both DNA and morphology with higher quality than either ethanol or formalin. Subsequently, DESS has been recommended as primary choice by a number of additional studies (e.g., Fonseca & Fehlauer‐Ale, 2012 on nematodes; Gaither, Szabó, Crepeau, Bird, & Toonen, 2011 on corals; and Ransome et al., 2017 on hard substrate communuties). These studies showed that DESS‐preserved samples yielded higher quantity and quality DNA compared to samples preserved in ethanol and RNAlater (Dawson et al., 1998; Ransome et al., 2017), as well as a larger yield of PCR products (Gaither et al., 2011; Kilpatrick, 2002). This may be due to the fact that DMSO enhances the uptake of nuclease‐inhibiting substances EDTA and NaCl in the cells of the samples, whereas ethanol dehydrates the samples, resulting in denaturation of proteins (Dawson et al., 1998). In addition, DESS is easy to use in subsequent DNA extraction, as it can be simply discarded after centrifugation after which lysis buffer can be added directly, whereas ethanol needs to dry first. The superiority of DESS over ethanol may therefore cause a shift in the predominant preservation method in the future.

### 3.3 Sample preprocessing

DNA extractions are usually not performed on raw bulk samples, as these samples contain large amounts of sediment or other inorganic and organic compounds, the samples are not homogeneous, and the amount of recovered biomass is often too large. The samples are therefore preprocessed before DNA extraction. This generally consists of separating the organisms from the inorganic compound, size fractioning and homogenization.

For soft sediments, it is important to separate the organic compound from the inorganic compound (i.e., the sediment), as general inhibitors present in the sediment can hamper the enzymes active in PCRs, possibly leading to false negatives. Sample separation is based on traditional methods used in morphotaxonomy, as separating the fauna from the matrix helps to isolate the species, and can be achieved by sieving (based on differences in particle size), decanting (based on density) and/or elutriation (based on a different response to a continuous water stream; Uhlig, Thiel, & Gray, 1973). Isolating fauna from the sediment is generally more effective for hard‐bodied fauna (e.g., Copepoda, Isopoda), because soft‐bodied fauna (e.g., Platyhelminthes, Polychaeta) can be easily damaged (Rumohr, 1999). Molluscs are an exception in the hard‐bodied fauna, especially when using decantation, as they are probably retained with the sediment due to their relatively high weight. This will introduce an important bias leading to the under‐representation of molluscs in the results. Incorporating extra steps to separate mollusc shells from the remaining sediment should be able to evade this bias. In addition, the successfulness of these methods depends on the sediment type: for example, decantation works well for coarse sand but is often only partially successful with muddy sediment (Aylagas, Mendibil, et al., 2016). Fonseca et al. (2010) and Fonseca et al. (2014) therefore also include a Ludox protocol to isolate meiofauna from fine silt. This method is based on the differences between the specific density of the sediment particles and the fauna present in the sediment and involves mixing the sample with a solution that approximates the specific density of the fauna, causing it to float, while the sediment sinks (Burgess, 2001). In our literature survey, most studies used sieving or a combination of sieving and decanting (63%), while 25% used a combination of sieving and a Ludox step, and 17% used both sieving, decanting and a Ludox protocol.

To detect whether organisms are retained after sieving and to accelerate the process of separating organisms from the sediment, staining is often used to colour the organic fraction. Fonseca and Fehlauer‐Ale (2012) showed that Rose Bengal inhibits amplification during PCR and, as the effects of other stains are not well known, it is generally recommended to avoid staining when samples are to be used for molecular analyses (Carugati, Corinaldesi, Dell'Anno, & Danovaro, 2015).

Even after separating the inorganic from the organic fraction, the sample composition is often heterogenic and detecting all species present would demand performing a DNA extraction on the entire sample. The amount of retrieved biomass is generally too large to make this feasible. Instead, the sample can be homogenized, and a well‐mixed subsample can subsequently be used for DNA extraction. The homogenization must be thoroughly done, as the subsample should be representative for the whole community DNA. Depending on the volume of the total sample, Aylagas, Mendibil, et al. (2016) recommend using a mortar and pestle for the homogenization of small samples and the use of a mixer or blender for large amounts of biomass. Other options include the use of an Ultra‐Turrax homogenizer, a smoothie maker or bead‐beating (Brannock & Halanych, 2015; Brannock, Waits, Sharma, & Halanych, 2014). The equipment should be thoroughly sterilized with bleach and soap between handling separate samples to avoid cross‐contamination.

A subsample from a well‐mixed bulk sample is assumed to contain the DNA of all species present. Larger animals and more abundant species, however, contribute more tissue to the homogenized biomass, which may cause a bias towards detecting larger or more abundant species, while smaller and rarer animals may remain undetected (Deagle, Clarke, Kitchener, Polanowski, & Davidson, 2018). This is a problem that is more pronounced in high‐diversity samples, as well as in samples with complex communities (composed of multiple individuals) compared to simple communities composed of a single specimen per species (Hollatz et al., 2017). Elbrecht, Peinert, and Leese (2017) showed that DNA metabarcoding of freshwater macroinvertebrate bulk samples sorted by size recovered 30% more taxa than unsorted samples. This emphasizes the importance of size fractioning before DNA extraction, especially when specimens differ in size by multiple orders of magnitude. Of the studies analysed in our literature survey, the hard substrate samples were almost always separated based on life style (sessile versus. motile), and the motile fraction subsequently fractioned based on size (e.g., Al‐Rshaidat et al., 2016; Leray & Knowlton, 2015). By contrast, soft sediment samples mainly targeted a specific size‐group (e.g., meiofauna), and therefore did not require additional size fractioning—but see Cowart et al. (2015) on significant differences between 0.5‐, 1‐ and 2‐mm mesh sizes. It is important to note here that while large species may contribute more to the total DNA, it is still possible for these larger animals to be under‐represented in the amount of amplified DNA relative to small organisms due to primer amplification bias (discussed later in more detail).

### 3.4 DNA extraction

A wide variety of DNA extraction kits are available on the market, as well as noncommercial methods such as CTAB (cetrimonium bromide) procedures and phenol–chloroform procedures. Our results showed that the DNeasy PowerSoil kit (Qiagen) was the most used method for sediment and hard substrate samples (Figure 5). In plankton and diet samples, there was more variation in the extraction methods that were used (amongst others the DNeasy Blood & Tissue Kit and noncommercial methods; Figure 5).

In a comparison of a laboratory‐based protocol and two commercial kits (Qiagen's DNeasy Blood & Tissue and PowerSoil kit) to extract DNA from nematode bulk samples, Dell'Anno, Carugati, Corinaldesi, Riccioni, and Danovaro (2015) found that commercial kits were preferable, as they consistently provided higher quality DNA. The results were also more reproducible, and the processing time was reduced. Between the two commercial kits, the DNeasy Blood & Tissue kit yielded a higher concentration than the PowerSoil kit. Similar results were found by Jeunen et al. (2019), who extracted eDNA from water samples using seven different methods. The DNeasy Blood & Tissue kit resulted in the highest DNA quantity, whereas the PowerSoil kit and a silica‐based protocol yielded the lowest concentrations. CTAB may also perform well in terms of yield, as shown in a comparison study by Schiebelhut, Abboud, Gómez Daglio, Swift, and Dawson (2017). However, the DNA extractions in this comparison were conducted on single ethanol‐preserved specimens, instead of community samples, and therefore probably did not contain inhibiting substances. Although the DNeasy PowerSoil kit results in lower amounts of DNA, it is still widely used for sediment samples, probably due to the effectivity of the kit in obtaining high‐purity DNA and removing the general inhibitors that sediments usually contain. Vasselon, Domaizon, Rimet, Kahlert, and Bouchez (2017), for example, extracted DNA from freshwater diatoms with four different commercial kits and a nonkit protocol and quantified not only the yield, but additionally estimated the number of inhibitors during the subsequent PCR. The noncommercial protocol yielded the highest concentration, but also showed significantly higher PCR inhibition. As only a small amount of DNA is needed in the PCR, the quality of the extracted DNA could be more important than quantity.

### 3.5 Marker selection

Deciding which DNA marker region to target is one of the key considerations when using DNA metabarcoding (Leray & Knowlton, 2016). A good barcode is taxonomically informative, meaning that the barcode needs to be able to distinguish between species (i.e., that the DNA region should mutate at the right rate). At the same time, conserved primer binding areas, or degenerate primers, are required for the primers to be able to attach to the DNA of all the organisms in the sample (Carugati et al., 2015). Many DNA regions have been suggested and discussions have elaborated on which is the most suitable (e.g., Andújar, Arribas, Yu, Vogler, & Emerson, 2018; Deagle, Jarman, Coissac, Pompanon, & Taberlet, 2014). The mitochondrial cytochrome oxidase subunit I (COI) is considered one of the standard markers, especially for metazoans, and is used in worldwide projects such as the International Barcode of Life Project (https://ibol.org/). Traditionally a 658‐bp fragment, the Folmer fragment, is targeted. However, the design of a shorter 313‐bp fragment (Leray et al., 2013), providing paired‐end overlap with the Illumina MiSeq sequencing platform, has resulted in a shift toward using this shorter fragment for bulk metabarcoding of marine metazoa. Aylagas, Borja, Irigoien, and Rodríguez‐Ezpeleta (2016) showed that the Leray fragment (using the mlCOIintF × dgHCO2198 primer pair) outperformed the traditional Folmer fragment (using the dgLCO1490 × dgHCO2198 primer pair), as the former primer pair retrieved a higher number of metazoan taxa benchmarked with morphological methods and in addition returned fewer unassigned reads. Other mitochondrial markers include the 12S rRNA gene and the 16S rRNA gene. These are more conserved than COI and have not been as widely used, resulting in a less‐complete reference database, but may nevertheless be good complementary markers (Devloo‐Delva et al., 2019). The nuclear 18S marker is as commonly used as COI. Like the 12S and 16S regions, it is more conserved than COI, making it easier to develop primers for these regions, but they have less discriminatory power at the species level (Creer et al., 2016).

Choosing a barcode or a primer set is finding the balance between a trade‐off: greater taxonomic coverage comes at the cost of taxonomic resolution. The organisms targeted in marine monitoring studies often range over different phyla, and the choice of marker therefore depends on the target taxa. Our literature survey for example showed that when only benthic meiofauna was targeted, either the 18S V1–V2 region or the 18S V9 region was chosen, whereas for benthic macrofauna different regions of COI were used (Figure 6). For studies focusing on eukaryotes in general the COI 313‐bp fragment, 18S V4 or other 18S regions dominated. In samples targeting micro‐eukaryotes and algae mainly different 18S markers were used and in zooplankton samples a range of different COI and 18S regions. 16S was predominantly used in fish samples, and the 12S marker was solely used in samples targeting fish. Finally, *rbcL*, *tufA* and 23S were only used in algal samples, as they are plastid markers.

Overall, only 28% of all studies in our literature survey used a COI fragment, whereas 59% targeted a hypervariable region of the nuclear 18S marker. However, studies comparing the performance of COI and 18S have shown that 18S generally underestimates the number of species due to poor species resolution (Leray & Knowlton, 2016). For example, in a zooplankton study, mitochondrial COI and nuclear 18S showed similar coverage across phyla, but COI resolved up to three times more at the species level (Clarke, Beard, Swadling, & Deagle, 2017). Similarly, Wangensteen, Palacín, Guardiola, and Turon (2018) found higher species resolution and more reliable species assignment with COI, and obtained four times more molecular operational taxonomic units (MOTUs) when COI was amplified compared to 18S. However, COI sequences also contained a higher percentage of unassigned MOTUs and were in addition unable to identify microscopic groups that were detected by 18S, such as Apicomplexa, Protalveolata and Foraminifera.

It is becoming increasingly clear that a multimarker approach, using several primer sets, rather than basing conclusions on a single marker, will result in a more reliable estimation of species richness (Alberdi, Aizpurua, Gilbert, & Bohmann, 2018). Yet, 75% of all studies analysed in our literature survey still only used a single marker, 18% used two markers and 7% tested more than two markers. In a study comparing the amplification success of five primer sets of different COI regions, Hollatz et al. (2017) found that three primer sets combined recovered up to 90% of the species in the artificially assembled communities, whereas the success from a single primer set ranged between 47% and 62%. It should be noted that using multiple markers also increases workload and costs. Whether the gain (i.e., better recovery of species richness) outweighs the costs (i.e., a lower number of samples with the same budget) will depend on the research question and aim of the study. A cost‐effective solution is to pool the amplified products of several primer pairs prior to indexing. Zhang, Chain, Abbott, and Cristescu (2018), for example, used four primers pairs (three COI regions and the 18S V4 region) in a single Illumina run to amplify zooplankton species in a mock community. Using a single marker and single primer pair, a maximum of 77% of the species were recovered. Using only the three COI fragments, 62%–83% of the species were detected, compared to 73%–75% when only the 18S fragment was used. Species recovery increased to 89%–93% if both markers were combined. This shows that for the optimal balance between taxonomic coverage and taxonomic resolution, multiple or combined markers with several primer sets need to be used.

### 3.6 PCR amplification

The two final steps in the process from sample to sequence—amplification by PCR and the sequencing itself—are both important sources of technical bias. During these processes, DNA molecules are randomly sampled, causing a stochasticity that has an especially great impact on the detection of rare sequences, and thus on the reproducibility of the dataset (Leray & Knowlton, 2017). In addition, the PCR cycling parameters need to be optimal for efficient amplification, because suboptimal parameters can affect taxonomic resolution and species recovery.

The annealing temperature is one of the most critical parameters of the PCR thermal profile. Finding the right temperature is often difficult. Choosing a temperature that is too low will result in amplification of aspecific products, while setting the temperature too high will reduce the yield and will therefore result in a bias as not all the wanted products are amplified. To overcome this problem, a touchdown profile is sometimes used instead (Korbie & Mattick, 2008). This profile starts with a higher annealing temperature in the initial cycles and decreases the temperature with each cycle. However, several studies have found that using a single annealing temperature actually yielded a higher number of zooplankton OTUs (Clarke et al., 2017) and a higher number of matches between morphology‐ and metabarcoding‐based results of benthic macrofauna (Aylagas, Borja, et al., 2016). Of the two single annealing temperatures compared—46 and 50°C—the lower temperature performed best for COI (Aylagas, Borja, et al., 2016). Similar results were found for bacterial communities: a bias towards amplification of taxa with less primer mismatches increased almost exponentially with increasing annealing temperature from 47 to 61°C (Sipos et al., 2007). A touchdown profile can likewise create a bias, as the high annealing temperature in the initial cycles probably increases preferential amplification (Clarke et al., 2017).

Our literature survey showed that overall, 80% used a single annealing temperature, 14% a touchdown profile and 6% two different annealing temperatures. The touchdown profile is predominantly used in combination with the Leray fragment (the 313‐bp COI region), as that is the thermal profile described in the original paper (Leray et al., 2013). Single annealing temperatures used for this fragment were either 55–56°C or 45–46°C. For the COI Folmer fragment, annealing temperatures below 50°C were used. To amplify 18S fragments, a single annealing temperature was used in all but one study. A temperature of 57°C was predominantly used for the 18S V9 and 18S V1–V2 regions, while 50°C was mostly used for the 18S V4 region. The optimal annealing temperature depends, among other factors, on the GC content of the primers, but also on the concentration of the primers and the DNA polymerase used (Nichols et al., 2018). This is especially important when using several primer pairs in one PCR. Doi et al. (2019), for example, used two primer pairs to respectively detect bony and cartilaginous fish with eDNA. They found that, when considering a range of annealing temperatures, the detection rate of bony fish was higher above 57°C, but cartilaginous fish detection was maximized at 58–59°C and decreased when the temperature was either higher or lower. This was probably due to differences in GC content of the primers. We are unable to cover all the physics of primer design and annealing temperatures in this review—Hollatz et al., (2017), Freeland, (2017) and Lobo, Shokralla, Costa, Hajibabaei, and Costa (2017) provide more detailed discussions.

To circumvent annealing issues with primers or when working with low amounts of DNA, the number of PCR cycles is often increased above 30. In our literature survey, the majority used 30 cycles or more (10%, 41% and 22% used 30, 35 and 40 cycles, respectively) and only 27% used fewer than 30 cycles. Although raising the number of PCR cycles does not affect the amount of mutations due to polymerase errors (Vierna et al., 2017), it may increase the formation of chimeras or increase amplification bias and has therefore been discouraged by Ahn, Kim, Song, and Weon (2012) and Aylagas, Borja, et al. (2016). However, other studies show that a reduction of PCR cycles has little impact on the results obtained (Krehenwinkel et al., 2017; Sipos et al., 2007).

To account for the stochasticity of PCR amplification, it has been strongly recommended to use at least three PCR replicates in bulk metabarcoding (Bourlat, Haenel, Finnman, & Leray, 2016). Yet, other studies argue that stochastic processes during PCR should have limited effects on bulk samples as those contain high amounts of DNA (as opposed to eDNA samples, which contain traces of DNA, and is therefore more subjected to stochastic events) and that increasing sequencing depth is more likely to yield robust results (Smith & Peay, 2014). In a bulk metabarcoding study of fungi in soil, for example, increasing PCR replicates up to 16 did not have a significant effect on species richness or turnover (alpha and beta diversity), whereas resequencing the same sample with increased sampling depth improved the reproducibility (Smith & Peay, 2014). A study on marine artificial mock communities likewise showed that stochastic events during sequencing contributes most to differences in presence–absence due to random sampling of rare taxa, while biases during PCR predominantly affect read numbers (Leray & Knowlton, 2017).

Of all the studies analysed during this review, 48% did not utilize PCR replicates, 23% of the studies used two replicates, 24% used three replicates and 5% used more than three PCR replicates. As with many of the methodological decisions that must be made during the research design, choosing the number of PCR replicates will depend on the research question or goal of the study. Increasing PCR replicates will also increase costs and laboratory time, so when finding the majority of species present in the sample will provide enough information (e.g., when doing ecosystem assessment studies), or when interested in using presence–absence data sets, increasing PCR replicates are not likely to improve the results. If the aim is to detect rare species (such as nonindigenous species in plankton samples), increasing sequencing depth is more important than using a higher number of PCR replicates. However, when the main goal is to obtain an as complete as possible picture of the biodiversity or when conclusions on changes in relative read numbers are important, using at least three PCR replicates could improve the obtained results and outweigh the costs. In studies using dietDNA, the target DNA is at least partly degraded and present in smaller amounts. Stochastic processes are therefore more likely to take place and dietDNA studies could likewise benefit from an increased number of PCR replicates. Alberdi et al. (2018), for example, studied the diet of bats using faeces samples and not only found substantial difference between PCR replicates, but also showed that the way the PCR replicates are processed in the bioinformatical pipeline significantly alters results. They recommend using a relaxed approach of restrictive PCR replicate filtering to find the optimal balance between additive filtering (leading to inflated diversity) and strict restrictive filtering (resulting in erroneously removed sequences).

### 3.7 Sequencing

Several high‐throughput sequencing platforms have been historically used in DNA metabarcoding studies. From those, IonTorrent, Roche 454 (no longer available) and Illumina are the most well known. The Illumina technology currently dominates the market (>65% of the studies analysed in this review), especially after the shutdown of the Roche 454 platform in 2013. Several studies found that sequence platform (Roche 454, Illumina MiSeq, Ion Torrent PGM and Ion Torrent S5) did not affect species recovery (Braukmann et al., 2019; Eckert et al., 2018), although sequences obtained by Illumina had a higher quality (Braukmann et al., 2019). By contrast, a recent study by Singer, Fahner, Barnes, McCarthy, and Hajibabaei (2019) on eDNA metabarcoding of seawater found a much higher diversity in samples sequenced with the Illumina NovaSeq than with the MiSeq. This was partly due to sequencing depth, as the former has a 700 times greater sequencing depth than the MiSeq. The same held true though even when the NovaSeq and MiSeq were compared at similar sequencing depths. Possible explanations for this include differences in hardware, signal processing software and flow cells (Singer et al., 2019). The authors conclude that a great portion of the biodiversity in samples could be missed while using the MiSeq at lower sequencing depths (Singer et al., 2019). Increasing the detected diversity by elevating the sequencing depth is especially important in samples with lower traces of DNA (e.g., dietDNA samples) or in samples with high diversity. In addition, sequencing depth has been proposed to mitigate PCR amplification and primer bias (Alberdi et al., 2018). However, the dissimilarity between PCR replicates likewise increases with sequencing depth, indicating a higher number of sequence artefacts that can erroneously inflate the detected diversity (Alberdi et al., 2018). Thus, simply raising the sequencing depth to high levels comes at the cost of accurately characterizing the diversity and requires adjusting the bioinformatic processing to the correct level of sequencing depth.

Although widely used, the above‐mentioned platforms have a limited read length and, associated with that, a limited taxonomic resolving power. The PacBio platform allows for obtaining longer reads, such as the 658‐bp COI Folmer fragment and beyond (Hebert et al., 2018). Very recently, nanopore sequencing using the Oxford Nanopore sequencing technology has also become an interesting alternative sequencing platform. In contrast to the widely used Illumina sequencing platforms, nanopore sequencing is able to sequence DNA molecules of any length, and as such it can provide a better resolution as longer fragments can be analysed (Johnson et al., 2019). This would allow for the use of a complete 18S marker, or an amplicon combining both 12S and 16S as a single marker. Obviously, for this to be efficient, primer pairs need to be developed that can cover such longer fragments. Compared to Illumina sequencing, the PacBio and Oxford Nanopore platforms both have a lower raw read accuracy. However, this lower read accuracy can be overcome by the longer fragment length, or by using bioinformatics approaches that generate a consensus sequence from multiple raw reads (Nijland et al, in prep).

### 3.8 Current practices at European North Sea institutes

The results of the literature review showed that a wide variety of methods have been used and are currently in use (Figure 2). As the publications included in this review span nearly a decade (2010–2019), it is not surprising that some methods are now outdated. An obvious example is the 454 Roche sequencing platform, which was used in 25% of the studies but is no longer available. To discriminate between outdated and current approaches, nine European institutes were asked to fill in a questionnaire related to the processing and molecular methods of marine samples. These results are summarized in the following section. Detailed answers to the questionnaire are given in Materials S2.

Similar to the results based on the literature, a wide variety of sample preprocessing methods were used. Of these methods, sieving—sometimes combined with Ludox protocol—were most used for soft sediment and plankton, and size fractioning was used for all sample types. Despite older and recent publications on the success of DESS, ethanol was still the most popular preservation method. However, several institutes were at the moment of the questionnaire performing pilot experiments comparing ethanol and DESS.

The majority of the institutes used the DNeasy Powersoil kit for DNA extraction, both for hard substrate and for sediment samples. The DNeasyBlood & Tissue Kit (Qiagen), the E.Z.N.A. Mollusk DNA Kit (OmegaBio‐tek) and Macherey‐Nagel kits were also used for plankton.

A wide variety of DNA markers were targeted, including 12S, 16S (V3–V4 region), different lengths of COI and 18S, and larger fragments or complete mitogenomes. Overall, the COI marker was predominantly used, due to the higher taxonomic resolution and the availability of a more extended reference database. Other considerations included the availability of primers in the literature or ease of designing primers, the use of markers in previous data sets for which work was continued, and how well the specific target taxa are amplified and resolved by each marker. Only one institute combined the use of 18S and COI in order to include both phylogenetic backbone and high resolution. PCR amplification profiles varied extensively, including the used PCR polymerase mix. Examples of used polymerases include the Promega GoTaq DNA Polymerase, KAPA HiFi HotStart ReadyMix, Phusion HiFi Polymerase and the Thermofisher PhireTissue Direct PCR MasterMix. In addition to the previously discussed technical biases introduced during PCR, the choice of polymerase may also influence the results obtained. A study on the diet of shrimps, for example, found that the Bioline MyTaq Red Mix yielded better results, as the Invitrogen Platinum Taq reaction was more susceptible to PCR inhibitors (van der Reis, Laroche, Jeffs, & Lavery, 2018). A study on eDNA from marine sediment further showed that polymerase choice affects both occurrence and relative abundance estimates, probably due to a preference of the polymerase for sequences with a specific GC content (Nichols et al., 2018). Some polymerase kits are also incompatible with certain nucleosides, such as the KAPA HiFi kit with primers involving inosine (Zhang et al., 2018). Biases due to polymerase choice have received relatively little attention in the literature and are not always reported in the methods section.

Finally, five out of nine institutes used PCR replicates (mainly three replicates, but experimenting with up to 12 replicates) and eight out of nine institutes performed sequencing on the Illumina platform. One institute fully used the Oxford Nanopore Technologies, with two other institutes experimenting with this platform as well (especially considering primer‐free approaches).

The most important points for improvement were the use of negative controls and positive controls. Whereas the majority of the institutes used negative controls with each PCR run, this was not implemented as standard amongst all institutes. Negative controls are an essential quality control check and can be easily implemented as they do not take away sequencing depth if actually negative (i.e., there is no loss for the biological samples). Positive controls consisting of mock samples, synthesized DNA or older samples were only implemented by a minority. For positive controls, one or two samples will probably be sufficient. They too provide an important quality check (i.e., checking and correcting for cross‐contamination between samples during amplification and library preparation, as well as detecting tag switching; Barbato, Kovacs, Coleman, Broadhurst, & de Bruyn, 2019). Positive samples should be chosen carefully and are ideally from a different ecosystem to avoid cross‐contamination between samples (Elbrecht & Steinke, 2019).

## 4 CONCLUSIONS AND RECOMMENDATIONS

Our results demonstrate a wide variety of methodological protocols that can be used in DNA metabarcoding of bulk samples (Figure 2). Technical biases can be introduced with almost every step and probably cannot be avoided in its entirety. This calls for an urgent need to incorporate harmonized protocols. To minimize the impact of the technical biases, however, and to obtain results comparable within and between studies, standard procedures should be followed. This is essential when implementing DNA metabarcoding in standard monitoring. We furthermore stress that standardization starts with reporting laboratory protocols and bioinformatical methods clearly and accurately. In addition, making demultiplexed raw data, OTU tables and taxonomy read counts available would greatly improve the reproducibility of each study.

Although fine‐tuning is needed for each specific sample or study design, we offer the following generic recommendations for best practice protocols and standard procedures for DNA metabarcoding of marine bulk samples:

- The sample methods naturally depend largely on the study design and account for biological variation more than technical variation. However, it is advised to specify the target organismal group before sampling, as this will influence further choices made.
- If staining is necessary for additional morphological analyses, it is recommended to split the samples before staining and use an unstained sample for DNA analyses.
- It is important to homogenize the samples thoroughly before subsampling and DNA extraction in order to obtain a subsample representing the whole community present in the larger sample. Homogenization is preferably done with a blender or smoothie‐maker for larger samples and a mortar and pestle or ultra‐turrax for small samples.
- DESS is the preferred preservation solution, as this results in the highest quantity and quality of DNA, is easy to use in the field even at remote locations and does not pose any restrictions on shipping.
- The Qiagen DNeasy PowerSoil kit is recommended, as previous studies showed that this kit is highly effective to obtain high‐purity DNA and to remove the general inhibitors. This is especially important for samples containing traces of sediments, but to minimize differences between protocols and therefore minimize biases, this extraction kit is also recommended for other types of bulk samples.
- In metabarcoding studies, at least COI (313‐bp fragment) should be used, and if needed 12S, 16S, 18S and/or longer fragments in addition. When the research aim is to discover as much of the biodiversity in a system as possible, using multiple markers (or deeper sequencing as an alternative) is strongly advised.
- The PCR thermal profile is one of the most bias‐sensitive steps throughout the process from sample to sequence, and results can only be compared across samples or studies if PCR protocols are strictly similar.
- The PCR thermal profile should preferably have a fixed annealing temperature, as touchdown protocols should be avoided.
- The number of cycles in the PCR thermal profile should preferably be fewer than 35.
- The advisable number of PCR replicates depends on the aim or research question of the study and whether the costs will outweigh the benefits. Also note that the way PCR replicates are processed further down the bioinformatical pipeline will have a marked impact on the results. For every sample of extracted DNA, it is advisable to use at least three PCR replicates when:
  1. The aim is to find an as complete as possible picture of the biodiversity.
  2. The analyses will take into account changes in relative read counts.
  3. Working with degraded or low‐input target DNA (i.e., dietDNA obtained from stomach/gut content or faeces).
Using zero PCR replicates will outweigh the benefits when
  1. Finding the majority of species present provides enough information (e.g., ecosystem assessment studies).
  2. The analyses will take into account presence–absence data frames.
- Include several negative controls and at least one or two positive controls. Including a positive control (e.g., a mock sample or synthesized DNA) can help to test for optimal bioinformatic parameters further down the pipeline, to define amplification efficiency, to calibrate for PCR amplification bias and to detect tag switching. Positive samples should be chosen carefully and are ideally from a different ecosystem to avoid cross‐contamination between samples.
- When increasing sequencing depth in order to detect a higher number of taxa, the number of artefactual sequences also increase. This has to be accounted for, such as by setting copy number thresholds, using relative copy number thresholds or by rarefying the data set to the lowest number of sequences that a sample contained.

## 5 CHALLENGES AND PERSPECTIVES

### 5.1 Current limitations

Despite the fact that the use of DNA metabarcoding has been clearly demonstrated in terms of costs and timewise efficiency (Aylagas et al., 2018), its implementation is limited in current monitoring programmes. This is, amongst others, caused by the following:

#### 5.1.1 Matching to morphology‐based methods

The first main bottleneck is the long tradition of morphologicallly based methods in existing monitoring programmes and ecosystem status assessments. Apart from technical biases that may limit the implementation of DNA metabarcoding in current monitoring programmes, or future improvements that may improve implementation speed, a link with historical monitoring is important to keep in mind. Many current monitoring efforts are part of time‐series and long‐term databases that are fully based on traditional morphological data. Were these monitoring campaigns to suddenly switch to metabarcoding, this would almost certainly cause a shift in the observed species composition. Incorporating DNA metabarcoding is therefore only considered if the results obtained with metabarcoding can be matched to the results obtained with traditional morphotaxonomy.

Benchmarking metabarcoding with morphological assessments has been applied in several marine studies, mainly targeting soft sediment samples. A notable result is that metabarcoding in the majority of cases outperformed morphotaxonomy concerning the number of taxa retrieved (e.g., Cowart et al., 2015; Lobo et al., 2017; Vause et al., 2019). DNA metabarcoding in seagrass sediment samples, for example, yielded hundreds to thousands of MOTUs compared to only 323 species morphologically identified (Cowart et al., 2015). Likewise, Vause et al. (2019) found 62 orders in sediment samples from Antarctica with metabarcoding compared to 37 orders based on morphology. Similar results were found for plankton communities (Abad et al., 2016; Deagle et al., 2018) and hard substrate samples (Pearman et al., 2016).

Whilst often finding more taxa, metabarcoding does not necessarily cover the full taxonomic composition found with traditional morphology. In a study on zooplankton for example, 34 species were detected with metabarcoding and 21 with morphology, but the two methods only had 12 species in common (Deagle et al., 2018). This means that using only morphology, 22 species would have been missed, but using metabarcoding as the sole method, none species remain undetected. Likewise, Lobo et al. (2017) found that metabarcoding soft‐bottom macrofauna communities yielded 21–26 species that could not be detected with morphology, but using morphology in addition resulted in an extra three to six species.

Differences between results obtained by morphology or metabarcoding may partly be attributed to erroneous taxonomic assignment (with either method) or the presence of parasites, cryptic species (i.e., species that look morphologically similar but are genetically different) and other nontargeted species that are hard to detect by eye. Metabarcoding, for example, does not only identify larger species belonging to Cnidaria, Porifera and Rhodophyta, but also detects small eukaryotic groups such as Syndiniophyceae, Mamiellophyceae and Bacillariophyceae that would have gone undetected when using visual inspection only (Pearman et al., 2016). In addition, metabarcoding has a high resolution for problematic taxonomic groups, as well as specimens at immature stages or specimens that are physically damaged (Abad et al., 2016; Deagle et al., 2018; Lobo et al., 2017), and often has a higher specificity to detect nonindigenous species (Sundberg, Obst, Bourlat, Bergkvist, & Magnusson, 2018). This shows that metabarcoding allows for inclusion of taxonomic groups in biodiversity analyses that were formerly excluded when using classic monitoring methods.

However, morphology‐based methods may currently still yield better results for the traditionally monitored taxonomic groups. Aylagas et al. (2018), for example, detected higher alpha diversity with metabarcoding when taking into account all taxa (including those belonging to Fungi, Metazoa, Rhodophyta and Heterokontophyta), but when focusing on metazoans only, morphology yielded nearly twice as many benthic macrofauna taxa as metabarcoding. Similarly, Dell'Anno et al. (2015) conducted a study targeting deep‐sea nematodes, but found that only 32% of the OCTUs (operational clustered taxonomic units) were assigned to Nematoda and a similar percentage of OCTUs belonged to nontargeted Fungi (29%). In this case, morphological analyses actually resulted in a higher number of nematode species. This is probably due to an incomplete or inaccurate reference sequence database, which is especially problematic for inconspicuous taxa our relatively understudied species, such as deep‐sea nematodes (Dell'Anno et al., 2015; Faria et al., 2018).

#### 5.1.2 Incomplete reference databases

The existence of a less than perfect reference database (both in completeness and in correctness) is a second major bottleneck. Two of the most commonly used databases are the Barcode of Life Data Systems (BOLD) (Ratnasingham & Hebert, 2007) and the NCBI GenBank (Benson et al., 2013). BOLD currently contains ~8 million sequences (accessed February 27, 2020), is well curated and subjected to strict quality checks, and contains a lot of metadata including photos. However, a large proportion of the data (~40%) is private and not freely usable, which greatly restricts accessibility. GenBank is considerable larger as it includes sequences of all genetic markers (but not all of which are usable as barcoding genes), and currently contains >216 million sequences (accessed February 27, 2020) but is also thought to be less reliable and more prone to contain erroneous sequences and annotations (Bidartondo, 2008; Harris, 2003). Despite these concerns, recent analyses have shown that metazoan identifications in GenBank are accurate with an error rate probably below 1% at the genus level and can therefore be used reliably (Leray, Knowlton, Ho, Nguyen, & Machida, 2019). The accuracy of both GenBank and BOLD does differ per taxonomic group, for example as GenBank outperformed BOLD for the identification of insect taxa, while both databases function equally well for plants and macro‐fungi (Meiklejohn, Damaso, & Robertson, 2019).

The curation of reference databases is an ongoing task and debates continue over which genes should be included or should receive priority, how to ensure accuracy of species identification, what metadata should be required and which species should be prioritized. A recent gap‐analysis for example showed that while freshwater vascular plants and freshwater fish have a high coverage in BOLD and GenBank, only 22% of the marine invertebrates on the European Register of Marine Species list were covered (Weigand et al., 2019). In addition, it showed that up to 50% of some marine taxonomic groups (e.g., Annelida and Nemertea) were only available in private data sets in BOLD. This raises questions on how to ensure open access data whilst simultaneously protecting data ownership.

Even without any technical biases, the availability of a well‐curated reference database is crucial and solving this limitation may increase the number of species that can be detected by metabarcoding. All sequences in BOLD are automatically submitted to GenBank and BOLD regularly mines sequences from GenBank, but not all records are fully synced. Improving cross‐referencing has been suggested to increase the usability of both databases (Porter & Hajibabaei, 2018a). Another solution is to combine different reference databases when assigning taxonomic identification to raw sequences (Macher, Macher, & Leese, 2017), but this is only possible when the data are freely available. We also emphasize that taxonomists are essential to obtain a well‐curated reference database and to enable DNA metabarcoding to become a standard.

#### 5.1.3 Quantitative correlations

The final main bottleneck is the lack of correlation between the proportion of reads and the original proportions of species in the sample. Establishing whether this quantitative correlation can be found has been the focus of many recent studies and in‐depth meta‐analyses (e.g., Deagle et al., 2019; Lamb et al., 2019). While some studies found a good correlation between the proportion of reads and species abundance (Aylagas et al., 2018), sometimes after transformation of the data (Rossel, Khodami, & Martínez Arbizu, 2019), the majority of studies did not (Hollatz et al., 2017; Leray & Knowlton, 2017) and there is still little consensus. A weak correlation was found between read abundance and biomass, but not with number of individuals (Lamb et al., 2019). This may explain why studies with low differences in size/biomass between sampled specimens do show a correlation between species abundance and read abundance (Rossel et al., 2019). Probably even more important than biomass are the biases introduced during PCR and primer bias (Nichols et al., 2018). In the future these might be circumvented using PCR‐free methods (see section on Future improvements). Other possible solutions include the use of mock communities to infer information on the quantitative signal in the study concerned (Lamb et al., 2019) and defining correction factors (Thomas, Deagle, Eveson, Harsch, & Trites, 2016).

Being able to quantify species abundance is often a requirement in biodiversity monitoring and the inability to use metabarcoding for this purpose could increase the hesitation to incorporate DNA metabarcoding in current monitoring programmes. Although being able to obtain relative abundance from sequencing data is often regarded as the holy grail of DNA metabarcoding, it is not always necessary. The detection of nonindigenous species, for example, simply needs confirmation of the presence or absence of these species. Likewise, certain ecosystem indices function well without inclusion of abundance data (Aylagas et al., 2014). Currently, DNA metabarcoding still has practical limitations. Some of these are inherent to the techniques used and are unlikely to fully disappear. In contrast, we expect limitations such as the lack of reference databases and the matching to current monitoring practices to be tackled in the coming years. Below we provide an overview of the improvements that can be expected over the next years.

### 5.2 Future improvements

DNA metabarcoding techniques are still improving and hold exciting prospects in the (near) future. The main improvements in DNA database taxon detection and identification that we foresee are: (a) the bioinformatics involved in processing raw sequences and obtaining taxonomic assignment; (b) improvement of reference databases and markers used; (c) removing the PCR step; and (d) using whole‐genome information.

#### 5.2.1 Bioinformatics for taxonomic assignment

This review has focused on the biases that can be introduced in the methodological process of obtaining raw sequences from biological samples. After obtaining the raw sequences, however, the bioinformatical approaches employed to obtain taxonomic assignment will also influence the recovered community. These bioinformatical aspects have been reviewed previously in more detail (e.g., Alberdi et al., 2018; Coissac, Riaz, & Puillandre, 2012; Liu, Clarke, Baker, Jordan, & Burridge, 2019) and we therefore confine ourselves here to a brief summary. Several bioinformatical pipelines and software packages are available to process raw sequences, including mothur (Schloss et al., 2009), qiime (Caporaso et al., 2010), usearch (Edgar, 2010), the open‐source alternative vsearch (Rognes, Flouri, Nichols, Quince, & Mahé, 2016) and the R‐package dada2 (Callahan et al., 2016). Post‐processing of raw sequences includes, amongst others, read assembly, setting a minimum sequence copy number threshold to remove artefactual sequences (Leray & Knowlton, 2017), detecting and removing chimeric sequences that may have formed during PCR (Cordier et al., 2018), sequence clustering (Pauvert et al., 2019), and choosing an approach or algorithm to assign taxonomy (Coissac et al., 2012). When the selected thresholds are too conservative, rare species are erroneously deleted, whereas overly liberal parameters will result in an inflated diversity. Alberdi et al. (2018), in a study on bat droppings, showed that the highest proportion of biases is introduced through setting the copy number threshold and sequence clustering threshold, with the number of species detected differing up to four‐fold depending on the chosen threshold (e.g., 300 species were detected with copy number threshold = 1, while only 75 species were detected with copy number threshold = 100), whereas the effect of chimera removal was very limited. Although clustering sequencing into OTUs using a fixed dissimilarity threshold was one of the most common approaches, the use of exact amplicon sequence variants (ASVs) is gradually increasing. ASVs can distinguish sequence variants with single‐nucleotide differences and thus resolve genetic variation on a finer scale (Callahan, McMurdie, & Holmes, 2017; Pauvert et al., 2019). With the continuous development of new methods and bioinformatical tools, metabarcoding is increasing in resolution and specificity.

As the bioinformatical choices can greatly influence the recovered biodiversity, the lack of a standardized bioinformatical pipeline thus increases the existing difficulty in comparing between studies. Setting firm thresholds in a standardized protocol, however, will not benefit the results and approaches will need to be customized to each data set, taking into account, amongst others, marker choice, study design and target taxa (Alberdi et al., 2018). Including a mock sample (positive sample) can not only aid to define amplification efficiency and calibrate for PCR amplification bias but can also help to test for optimal bioinformatic parameters further down the pipeline.

#### 5.2.2 Improvement of reference databases and markers used

The second great improvement in DNA metabarcoding will be the vast expansion of the reference databases. Not only will more and more species be included, we expect that complete mitochondrial genomes and full‐length nuclear ribosomal sequences (18S–5.8S–28S) will be added instead of only short single markers. The rapid decrease of sequencing costs and the maturation of long‐read sequencing technologies such as nanopore sequencing will enable this transition. Importantly, not only will the reference databases obtain more completeness and better coverage of all species, but the metabarcodes themselves could become longer, providing higher resolution. Again, present and future developments in long‐read sequencing technology will enable this. The obvious advantages can already be seen in the microbial field, where the switch from short regions of the prokaryotic 16S ribosomal marker to full‐length 16S enables the metabarcoding resolution to move from the genus level to species and even strain level (Johnson et al., 2019).

#### 5.2.3 Removing the PCR step

Some technical biases are more important than others, and amplification by PCR caused by, for example, primer biases is one of the largest sources. Direct sequencing of mitochondrial DNA and/or ribosomal genes without amplification is the obvious solution. This could be achieved through target enrichment strategies enriching the DNA pool before sequencing. The recent development of adaptive sampling approaches is highly interesting in this context. Adaptive sampling or read‐until approaches allow for a short fragment of the DNA being read by the sequencer, and the DNA molecule is only sequenced completely if it is classified as being of interest for the particular experiment (Kovaka, Fan, Ni, Timp, & Schatz, 2020; Payne et al., 2020). This technique allows for implementing an enrichment step during the sequencing process. For biodiversity assessment, enrichment could be aimed at only the DNA fragments containing data required for taxonomic assignment. If these techniques mature, it will become possible to skip the amplification step and sequence the isolated DNA directly, removing a large source of bias from the DNA metabarcoding analysis. The relevance of sequencing longer fragments and full mitogenome or ribosomal gene clusters will be more pronounced for bulk sample DNA compared to dietDNA and especially eDNA, because in the latter samples most DNA fragments will have been degraded.

#### 5.2.4 Whole genome data

Currently, sequencing technology and data processing are becoming cost effective enough to omit specific amplification or enrichment, and instead just sequence the DNA from the sample directly. Again, for this to result in an effective taxonomic assignment, the reference database will be the major bottleneck. In this context the development of shotgun metagenomics approaches based on genome skims are worth mentioning. Here, the genome of target organisms is sequenced with a very low coverage depth (~0.5×). Subsequently, long reads obtained from the samples are compared to this incomplete reference database, allowing for many matches to the partially sequenced genome (Peel et al., 2019). This version of the shotgun metagenomics approach allows for the use of incomplete whole genome sequencing at much lower costs, while already taking advantage of the information present in the full genome. Eventually, the databases will mature, and complete genomes will be added for more and more species, enabling shotgun metagenomics to take over the metabarcoding strategies. Bulk metabarcoding is the perfect application for this, as there is relatively little DNA present in the samples which is not relevant for the biodiversity assessment of the sample (in contrast to, for instance, eDNA).

### 5.3 Metabarcoding as a complementary or stand‐alone method?

The key question related to metabarcoding is often whether we can use metabarcoding as a stand‐alone method to monitor biodiversity. To answer this question, it is natural to compare the results to those we would have obtained using traditional methods (see section on Current limitations). Although we can certainly learn a lot about the effectiveness of both metabarcoding and morphotaxonomy by making these comparisons, using morphology to “benchmark” whether metabarcoding provides a true estimate of alpha diversity may provide an incorrect contrast.

First, it is easy to forget that morphology—like metabarcoding—has biases as well. For example, classic monitoring of biodiversity often focuses on well‐defined groups (i.e., meiofauna or macrofauna in sediment samples, which requires sampling methods that in itself are biased), while excluding species that are harder to detect. However, these organisms may have an enormous impact on the ecosystem (e.g., fungi, parasites, phytoplankton and bacteria), or indeed may be key to functioning of these ecosystems and would be well worth including in monitoring programmes. In addition, by using well‐framed but limited groups as a measure of biodiversity, classic monitoring methods actually provide an underestimation of true alpha diversity as we currently accept it (V. G. Fonseca et al., 2010; Lobo et al., 2017) and is as such not appropriate to benchmark another method. Thus, metabarcoding leads us to think about biodiversity and species richness in other terms, and on a whole different scale again.

At the same time, a one‐to‐one comparison of morphology and metabarcoding is problematic, as the concept of “species” can often not be literally translated to unique sequences. Several concepts of molecular species are used across metabarcoding studies, including OTUs, MOTUs, OCTUs and ASVs and one taxonomic unit does not always correspond directly to a single morphological species (Blaxter et al., 2005; Brown, Chain, Crease, Macisaac, & Cristescu, 2015). By using metabarcoding, we may have to rethink the concept of “species” again. However, both morphological species and taxonomic units can be used to calculate ecosystem assessment indices, such as AMBI (AZTI Marine Biotic Index). The latter is based on the pollution tolerances of species present in soft‐bottom communities (Borja, Franco, & Pérez, 2000) and has been shown to perform equally well, despite differences in alpha richness estimates, when derived from either genetic or morphological data (Aylagas, Borja, et al., 2016; Aylagas et al., 2018). Therefore, the choice of whether to use metabarcoding as a stand‐alone method or as a complementary method depends on the goal of the study. Either way, it is clear that metabarcoding provides a valuable tool to monitor marine life that has been increasingly used in recent years and it is likely that its role will only increase in the near future.

## AUTHOR CONTRIBUTIONS

Luna van der Loos performed the research, Luna van der Loos and Reindert Nijland designed research, analyzed data and wrote the paper.

## Supporting information

Table S1

Supplementary Material

## Data Availability

The data collected for the analyses in this review are available in the online Materials S1 and on Figshare (https://doi.org/10.6084/m9.figshare.12768071.v1; van der Loos & Nijland, 2020).
