# Supplementary material for: Biases in bulk: DNA metabarcoding of marine communities and the methodology involved
Source: Mol Ecol. 2020 Aug 29;30(13):3270–88. doi: 10.1111/mec.15592 (PMC8359149; doi:10.1111/mec.15592)
Supplement: Supplementary file 2 — Supplementary Material [file MEC-30-3270-s001.docx]

**Supplemental Materials 2**

Supplemental information for:

**Biases in bulk: DNA metabarcoding of marine communities and the methodology involved**

Luna M. van der Loos^1,2^, Reindert Nijland^1^

^1^ Marine Animal Ecology group, Wageningen University, P.O. box 338, 6700 AH Wageningen, The Netherlands

^2^ Present address: Department of Biology, Phycology Research Group, Ghent University, Ghent, Belgium

Corresponding author: Reindert Nijland, email: reindert.nijland@wur.nl

**Content:**

1. Overview of the questionnaire
2. Overview of the responses to the questionnaire
3. **Overview of the questionnaire:**

Nine institutes involved in the EU Interreg North Sea region project ‘Genetic tools for Ecosystem health Assessment in the North Sea region’ (GEANS) were asked to fill in a questionnaire focusing on the methods they currently employ in bulk metabarcoding of marine samples.

The questionnaire is given below:


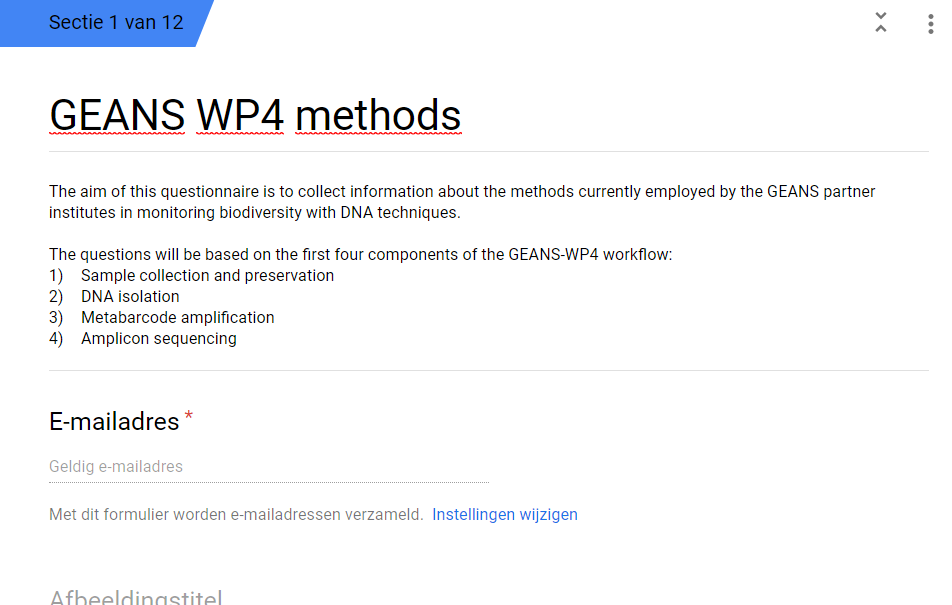


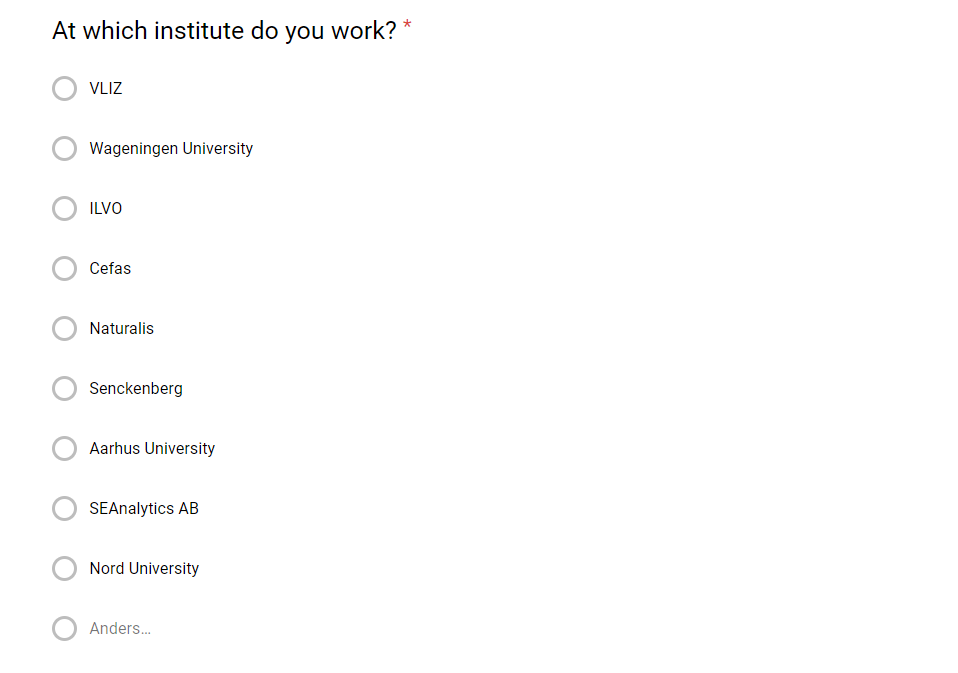


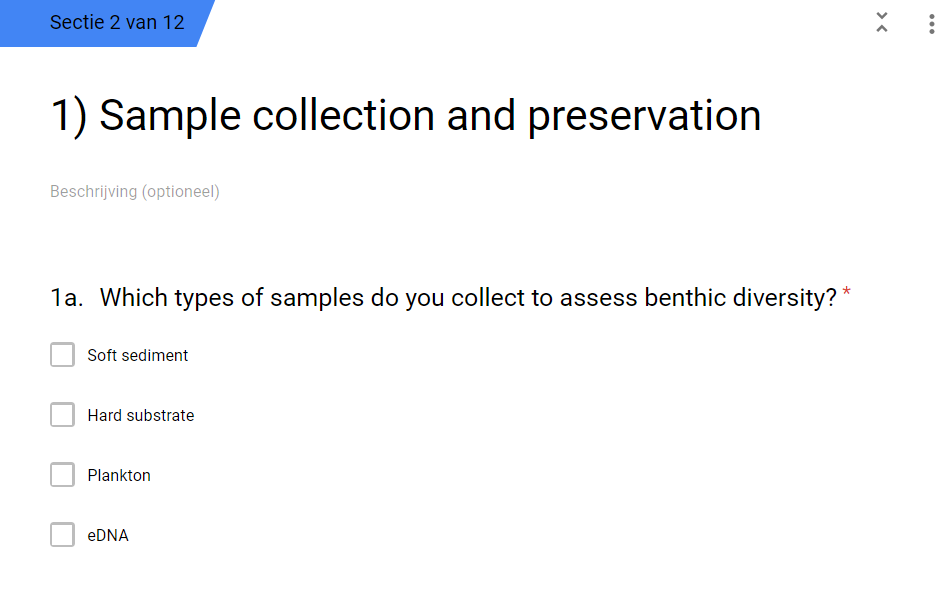


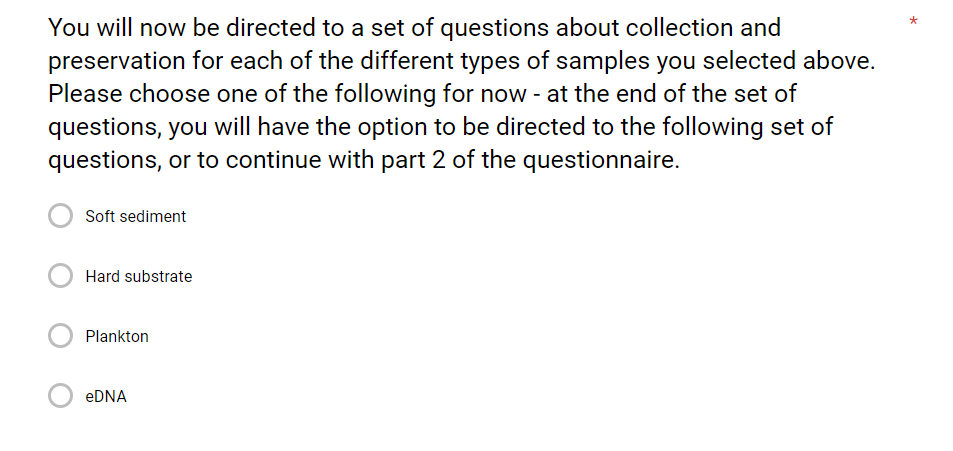


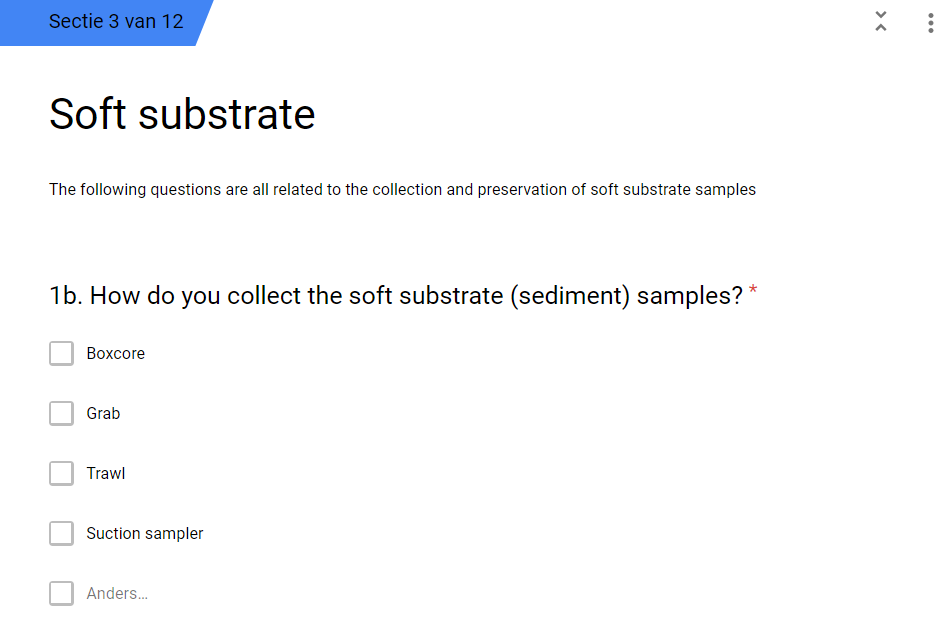


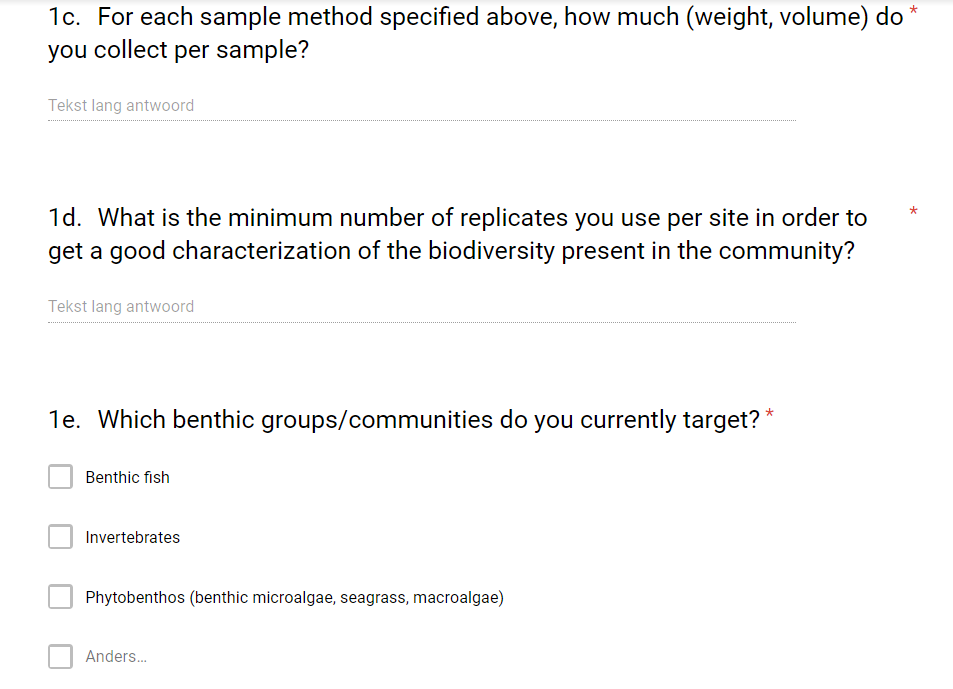


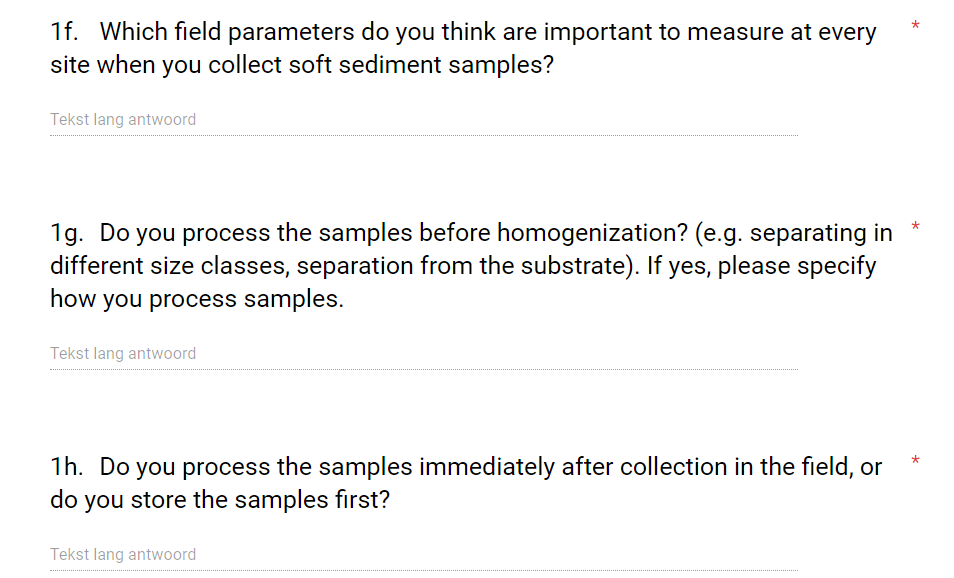


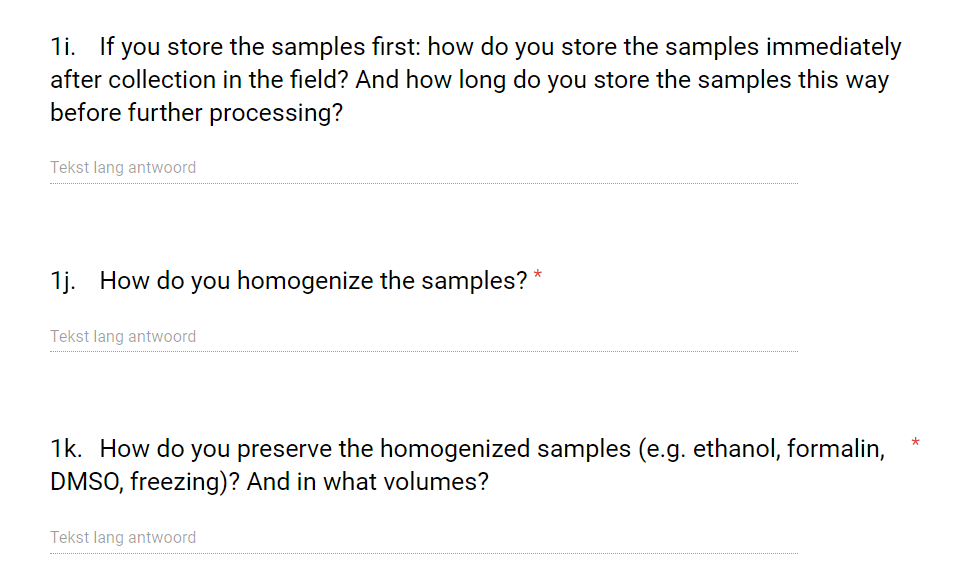


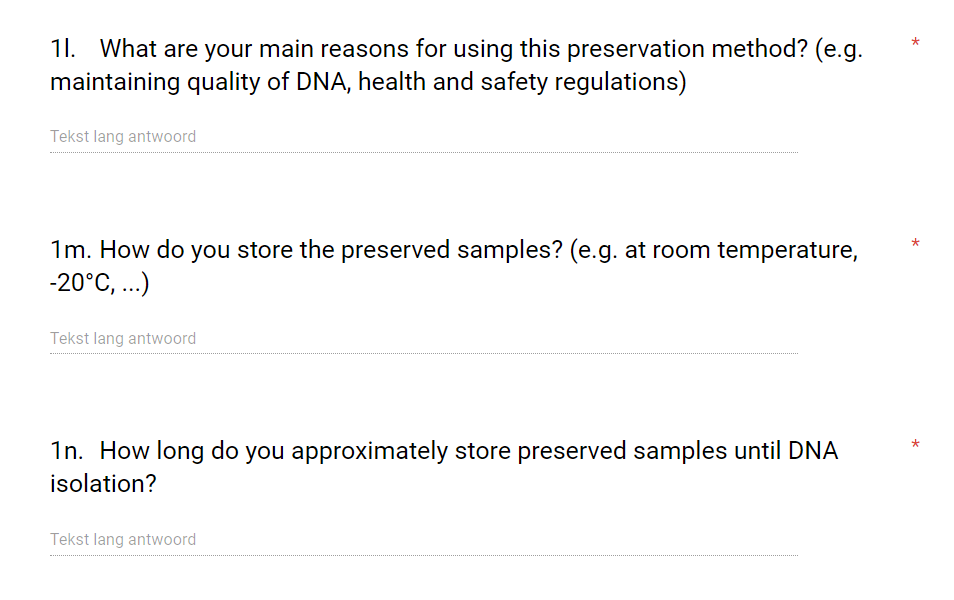


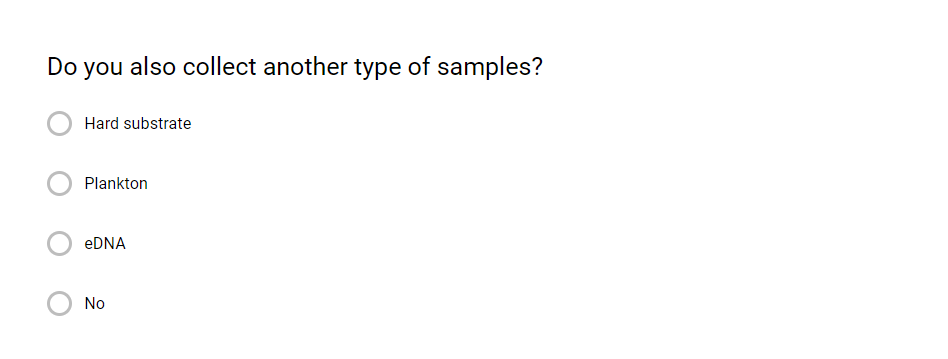


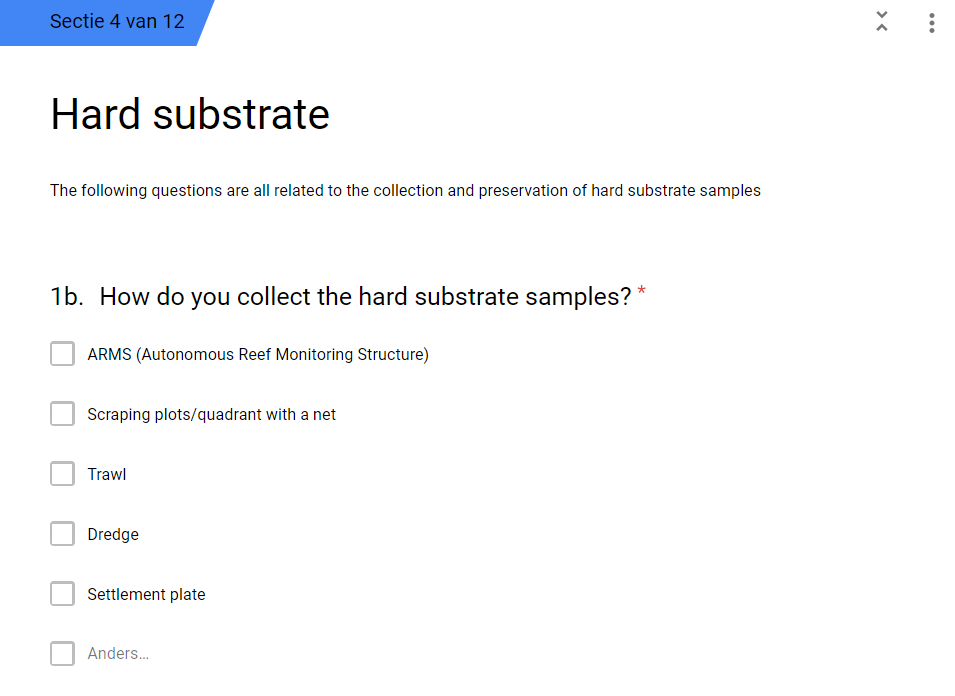

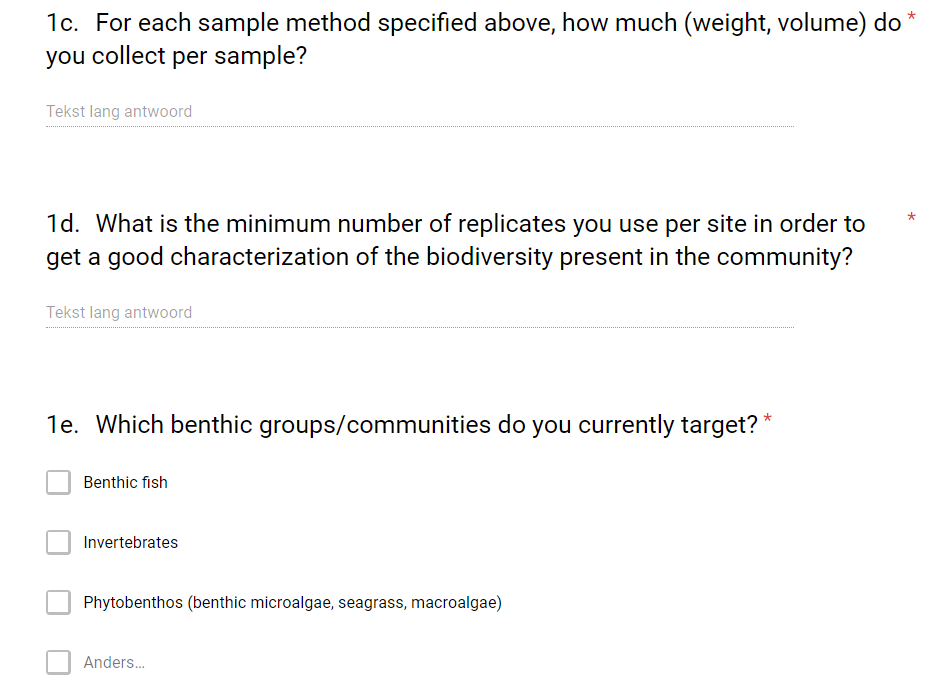


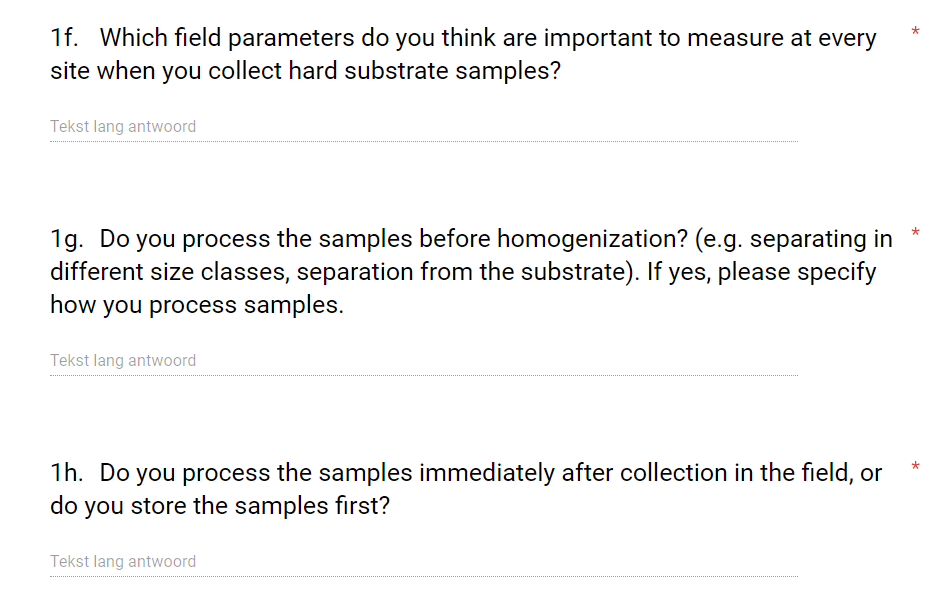

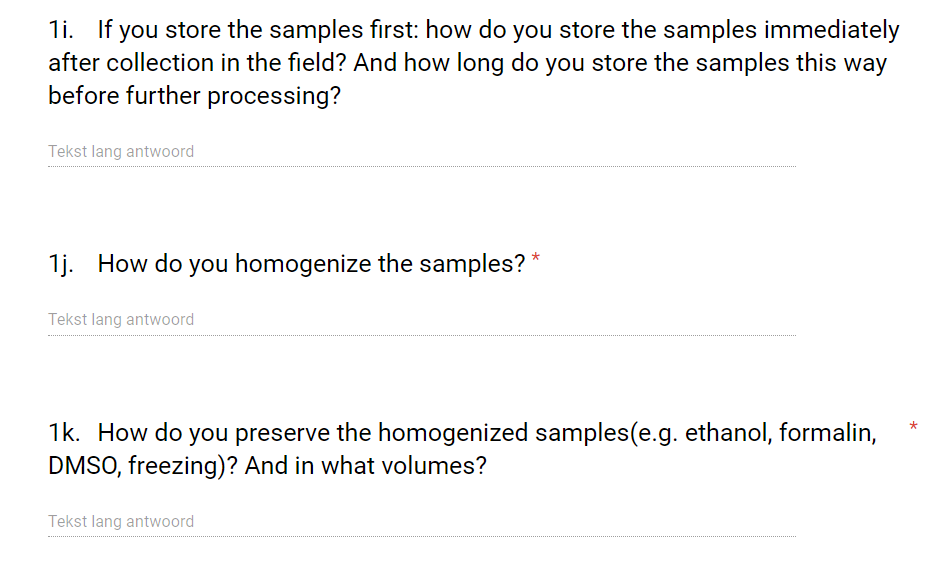


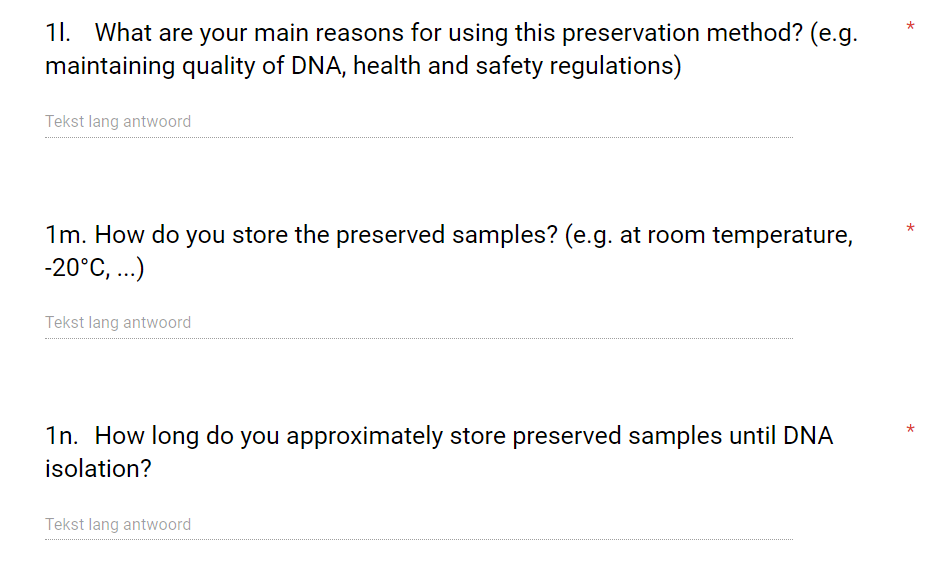

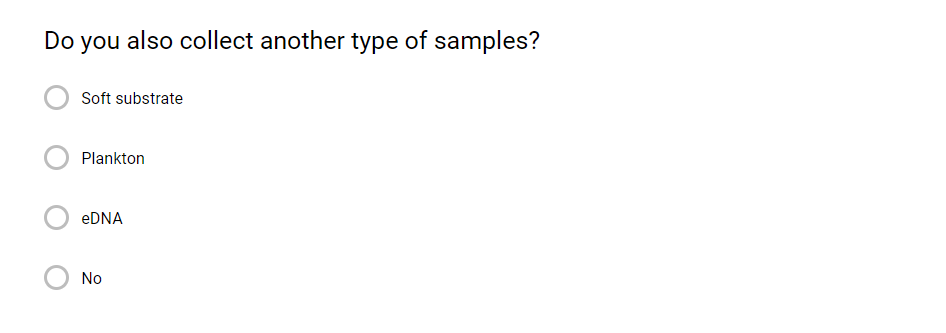


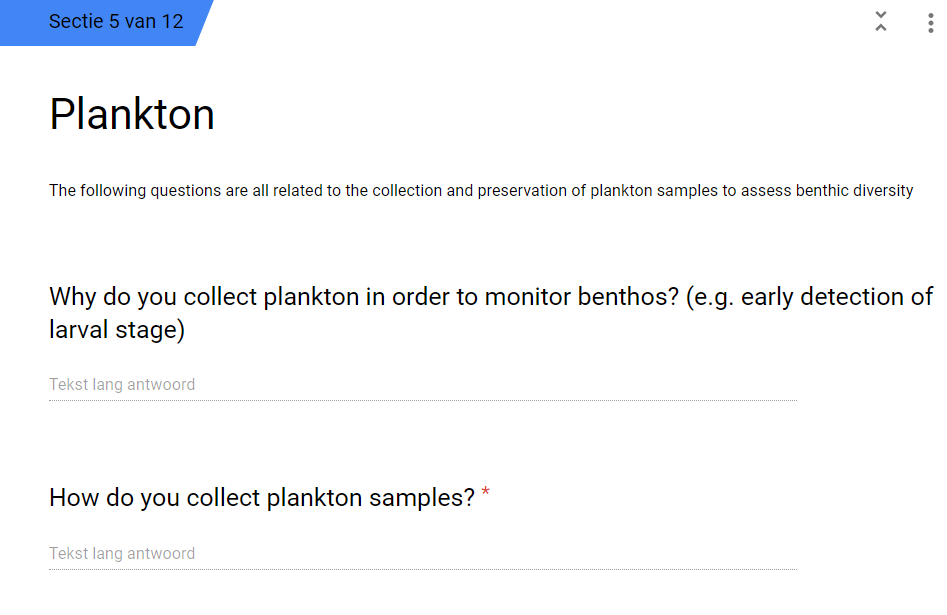

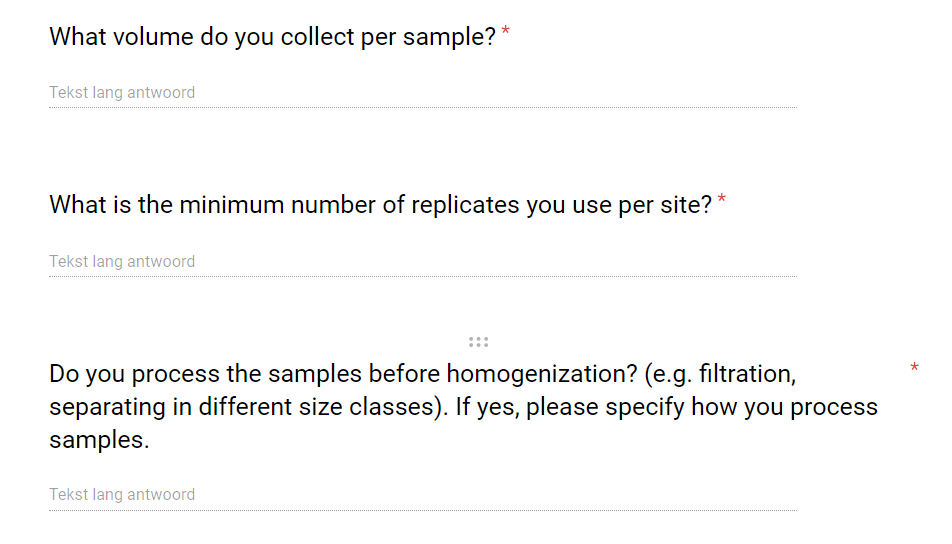


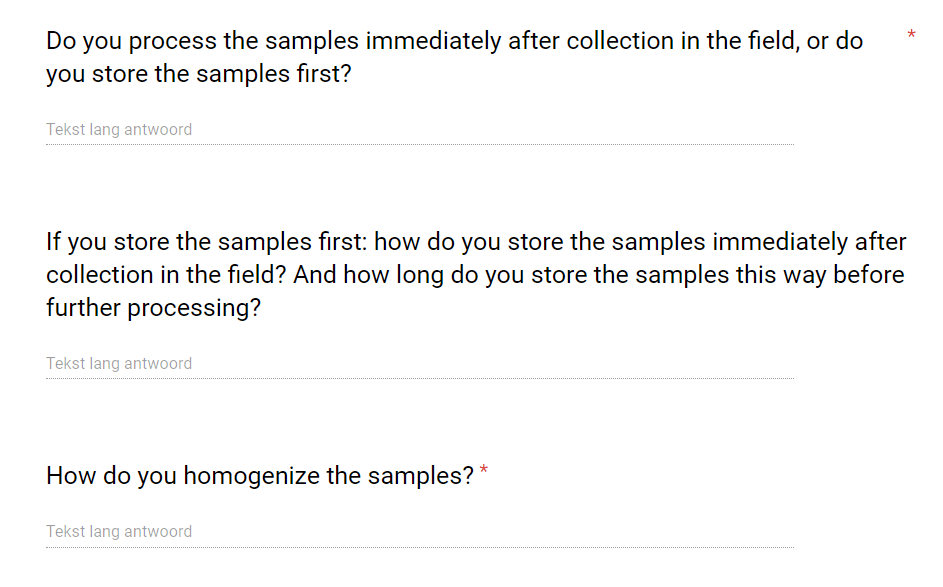

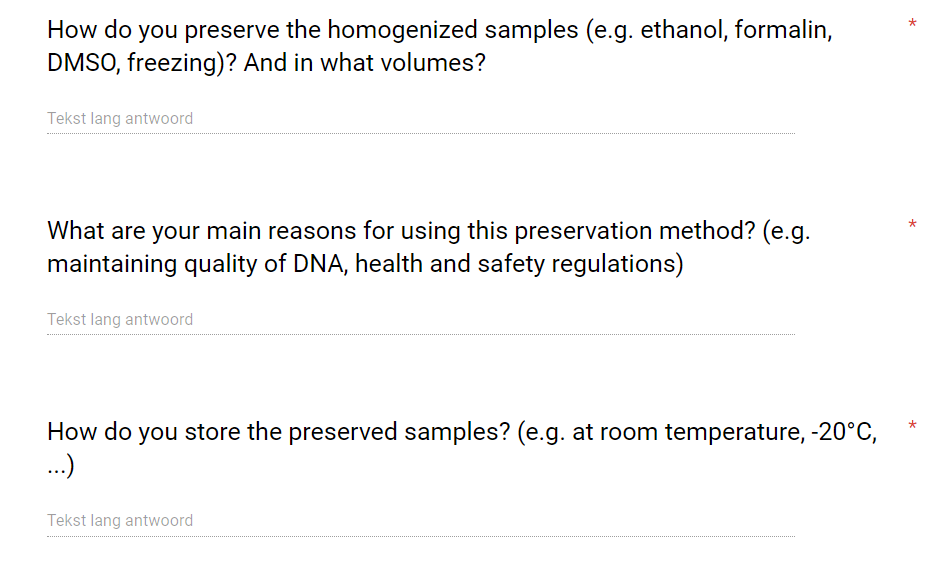


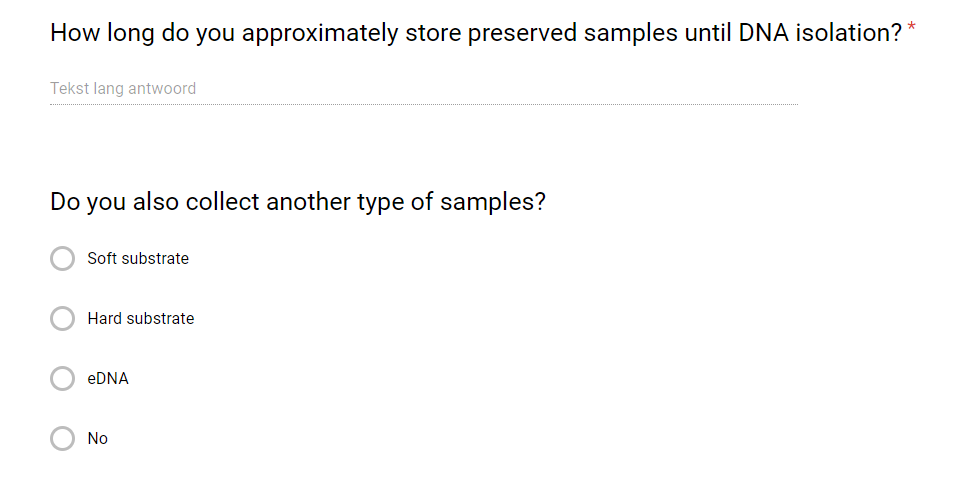


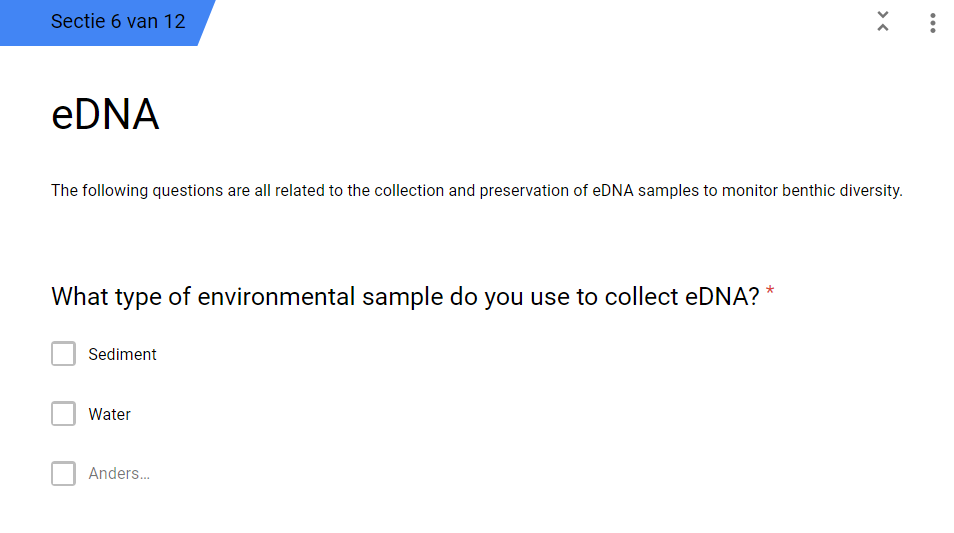


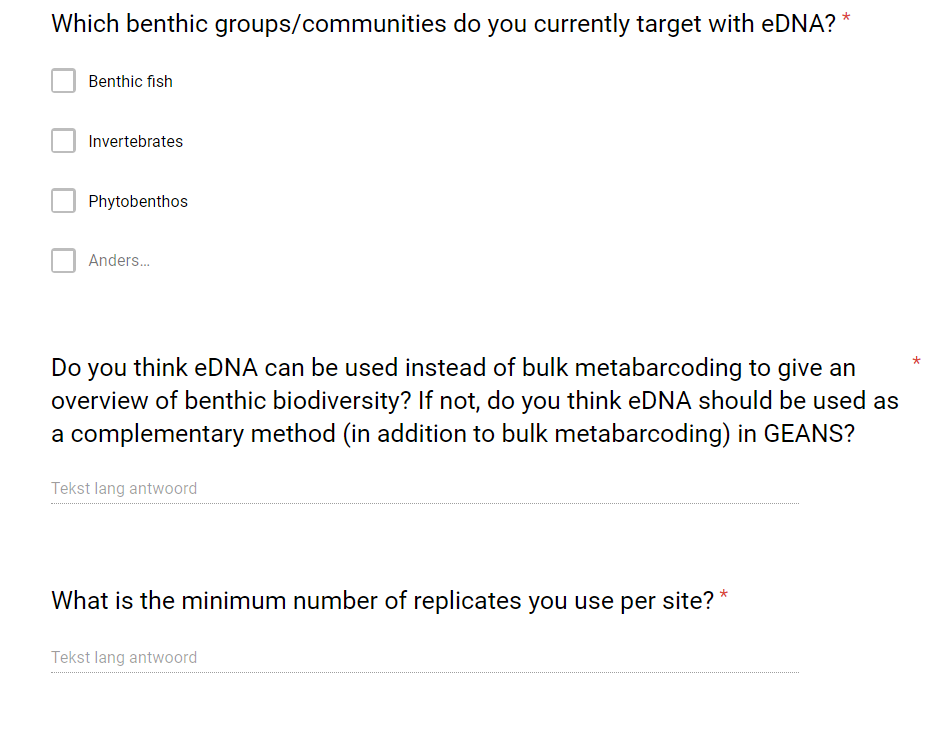

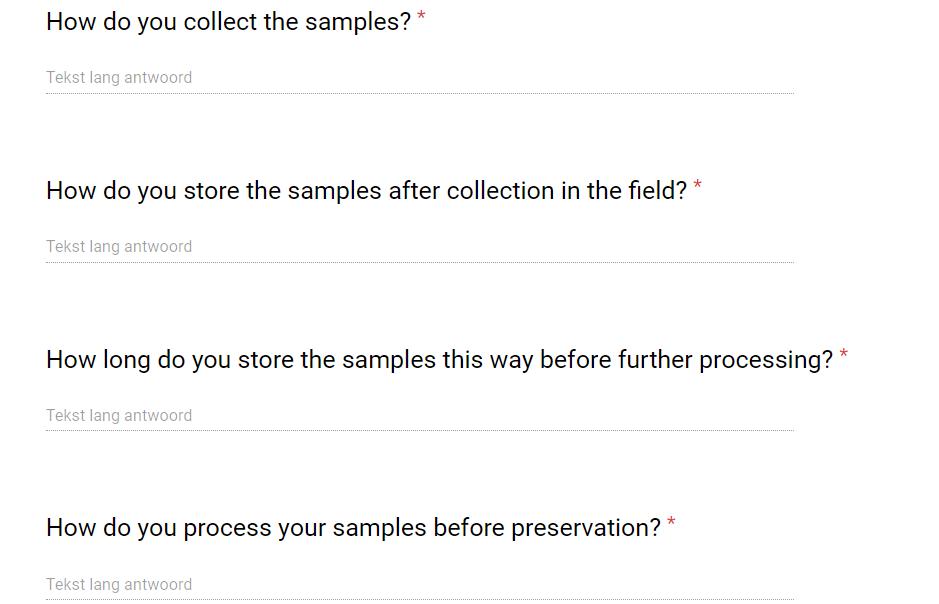


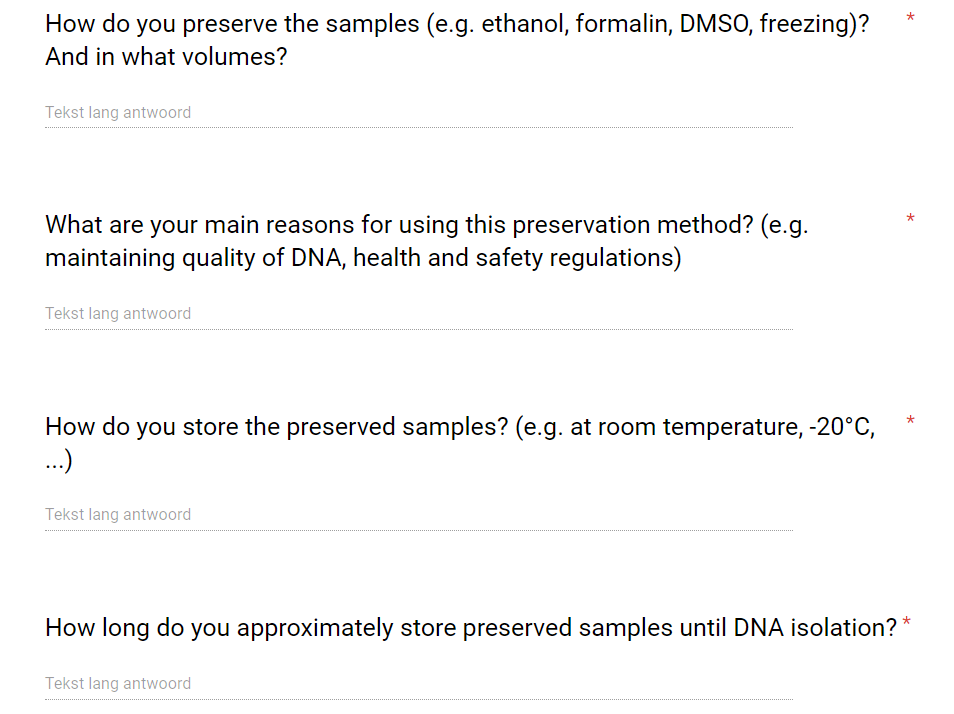


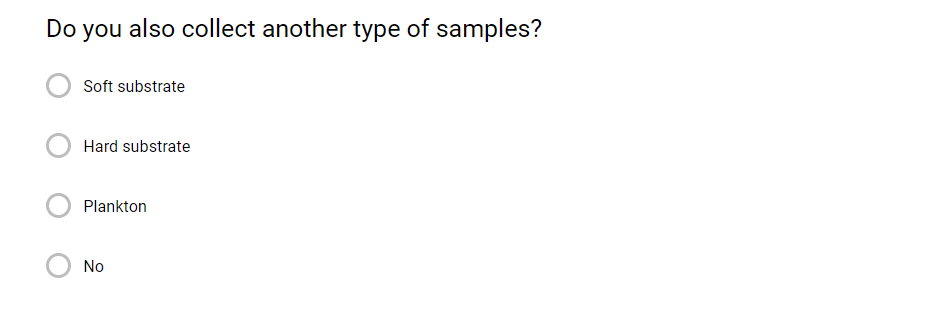


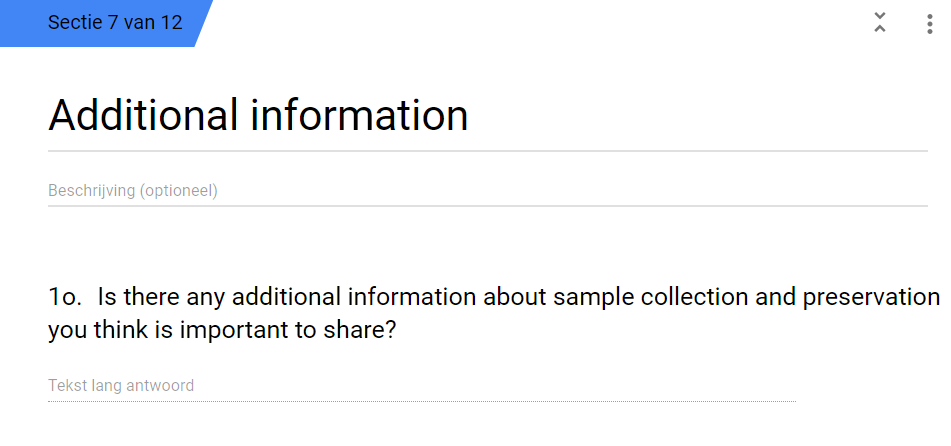


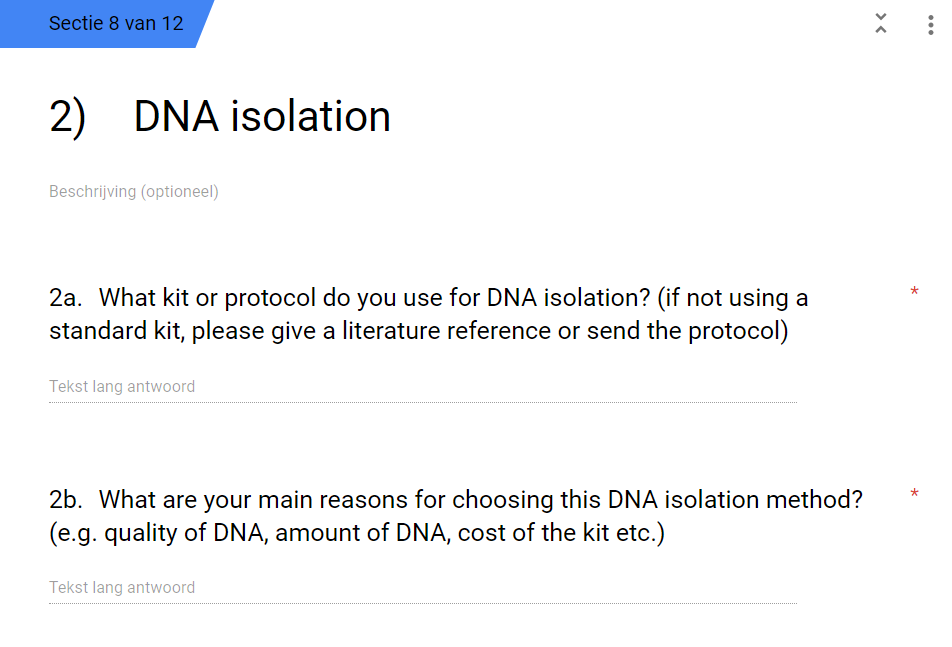


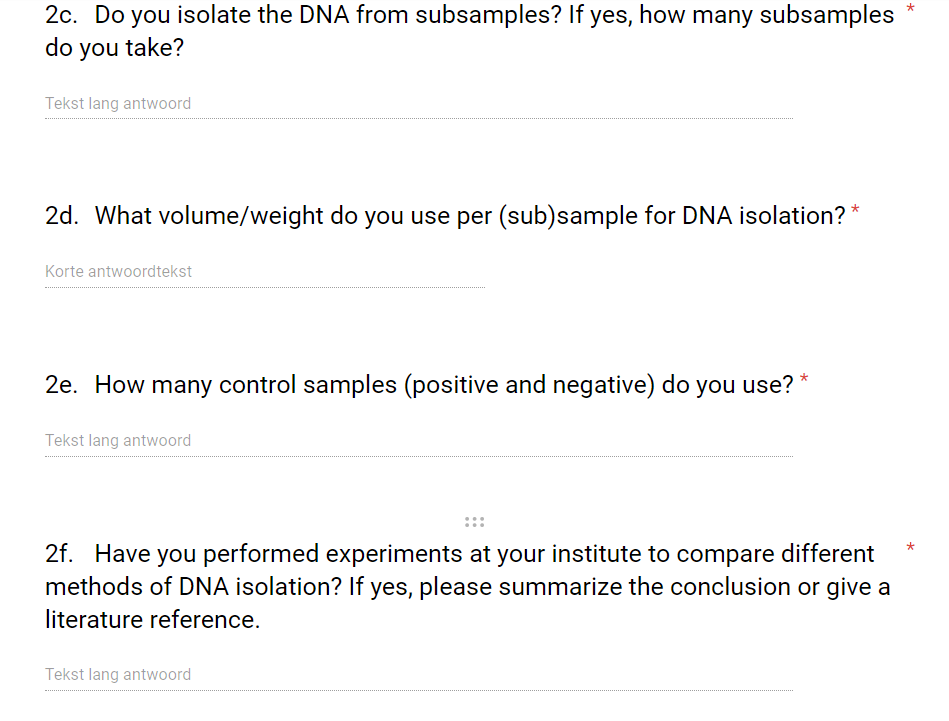


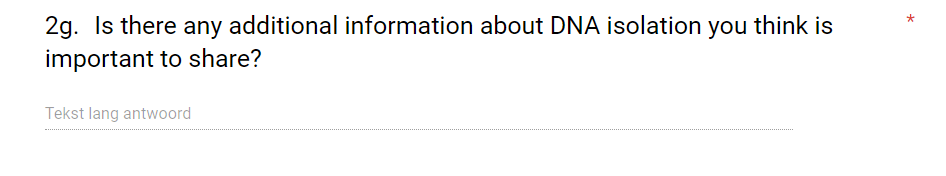


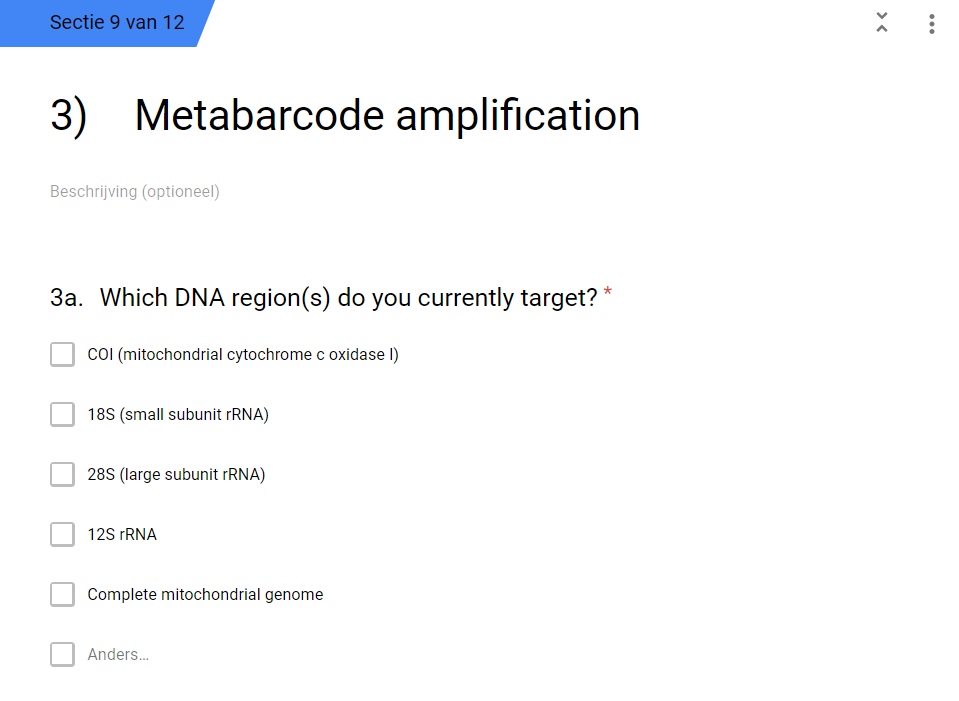

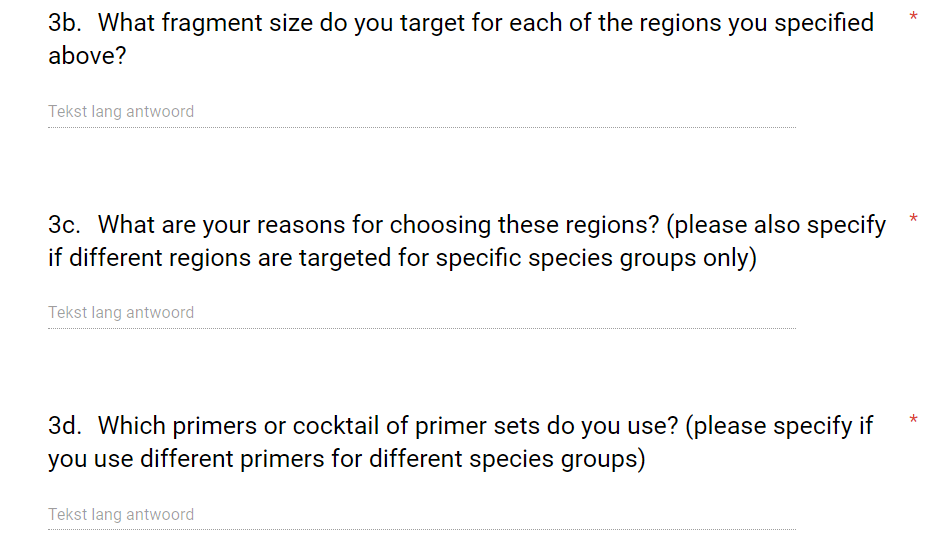


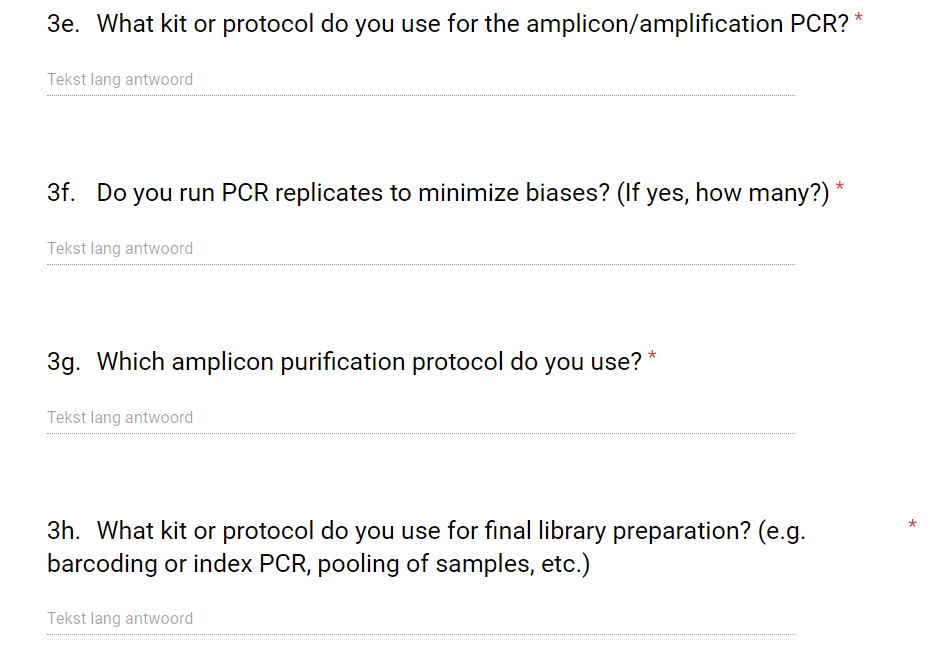


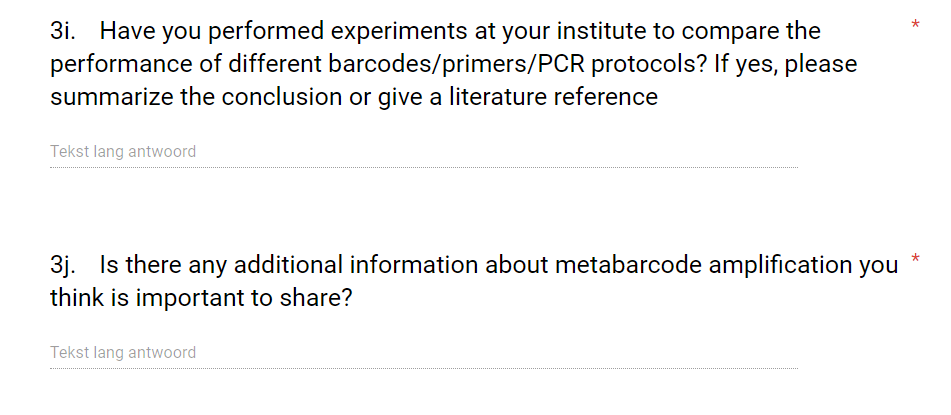


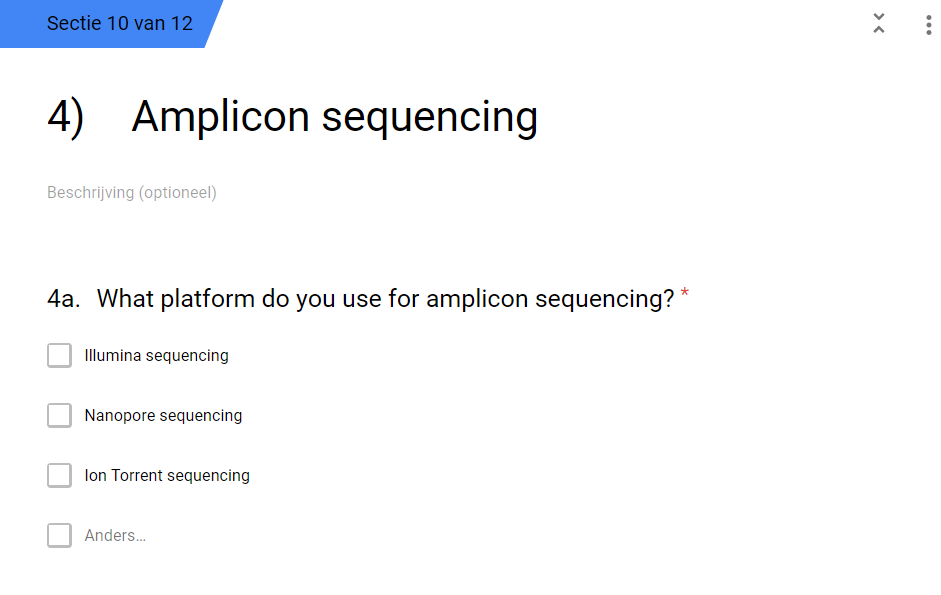

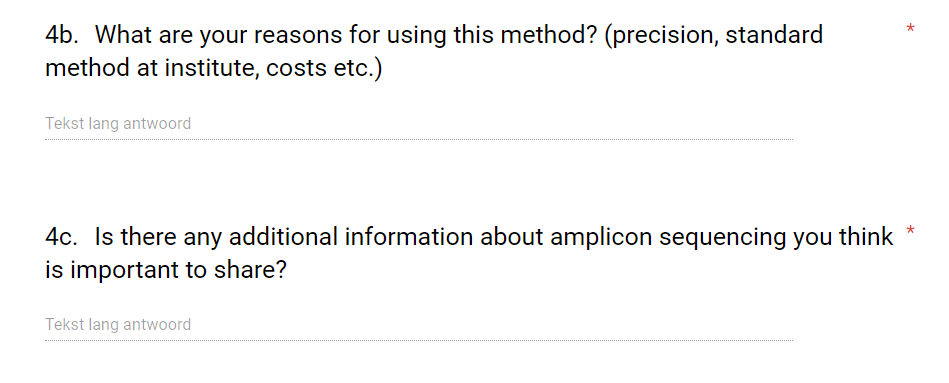


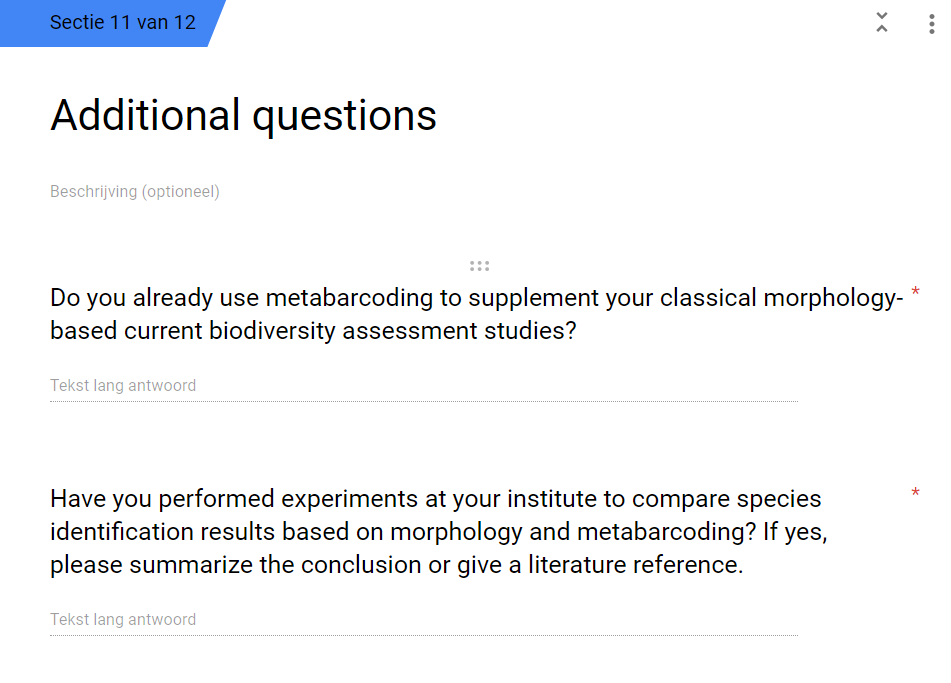


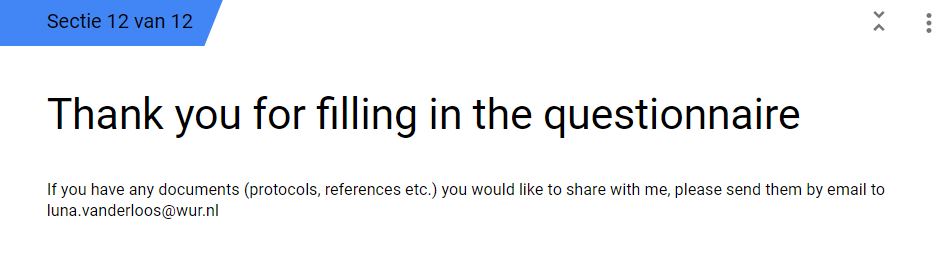


1. **Answers to the questionnaire:**

Nine institutes involved in the EU Interreg North Sea region project ‘Genetic tools for Ecosystem health Assessment in the North Sea region’ (GEANS) were asked to fill in a questionnaire focusing on the methods they currently employ in bulk metabarcoding of marine samples.

The anonymized responses to questionnaire are given below:


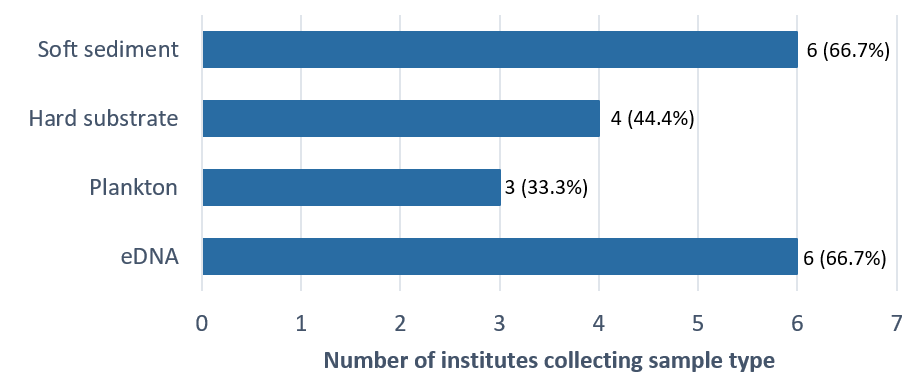


**Figure S1.** The number of institutes that collect different sample types, with the percentage of all GEANS partner institutes in brackets (e.g. soft sediment is collected by 6 institutes, which is 66.7% of all GEANS partners).


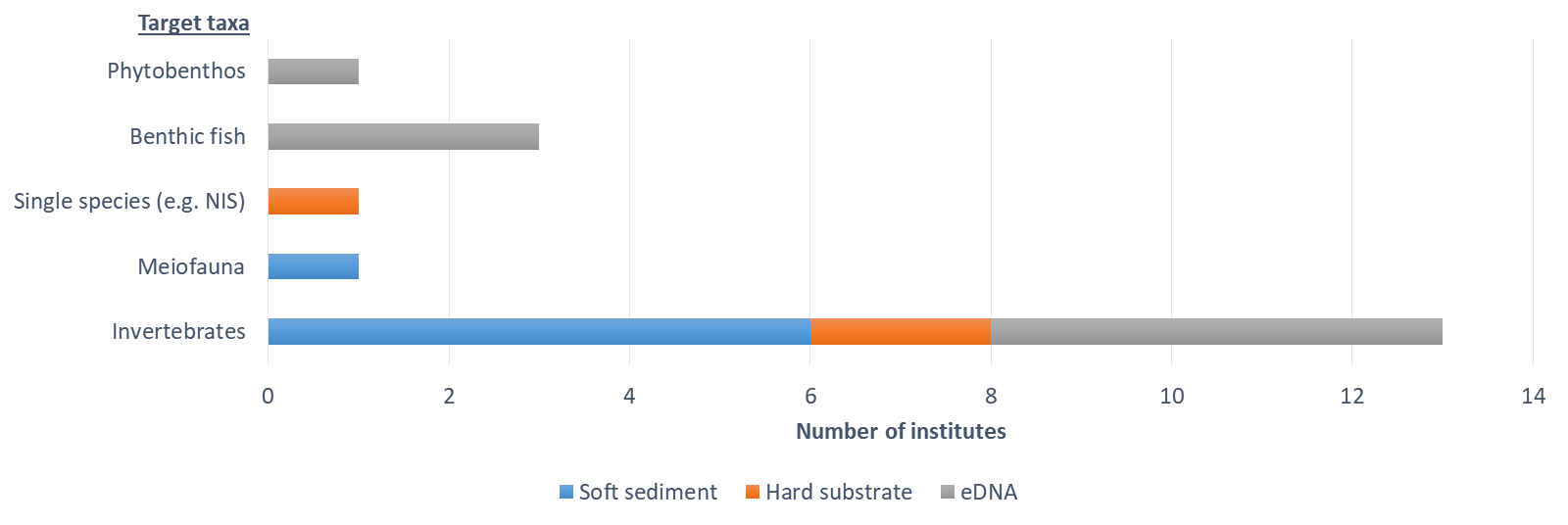


**Figure S2.** The number of institutes that target a specific taxa group (i.e. phytobenthos, benthic fish, single species, meiofauna or invertebrates), split into sample type (blue = soft sediment, orange = hard substrate, grey = eDNA).


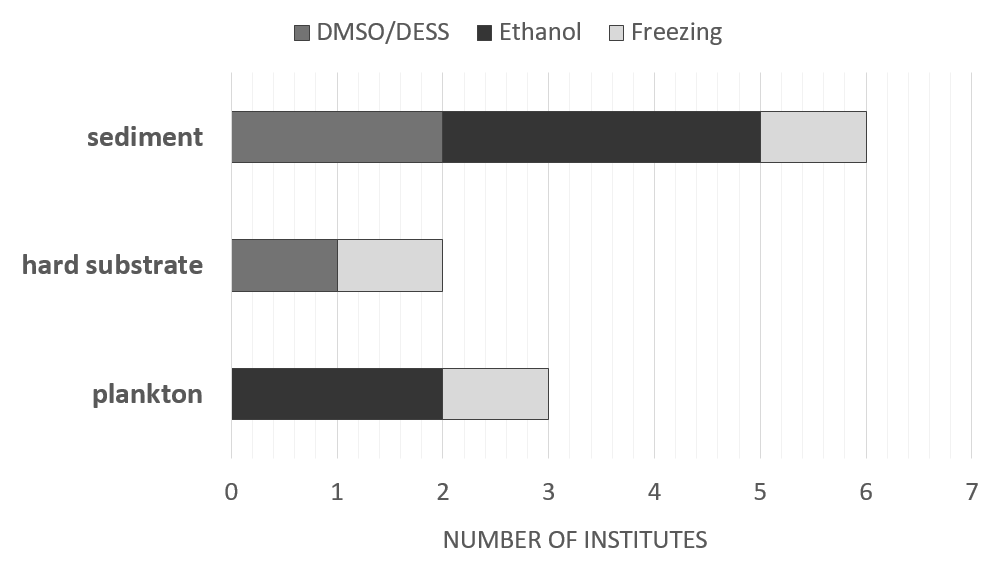


**Figure S3.** The number of institutes using a specific fixative (DESS, ethanol or freeze-drying) for sediment, hard substrate or plankton samples.


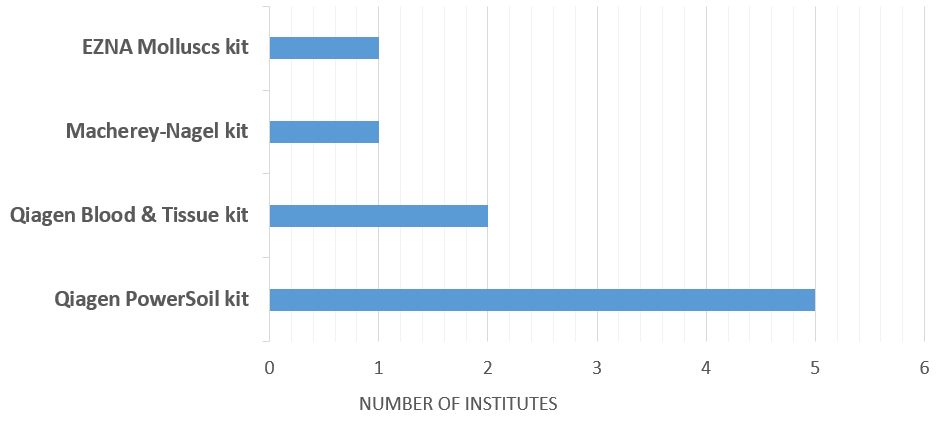


**Figure S4.** Number of institutes using a specific DNA extraction kit.


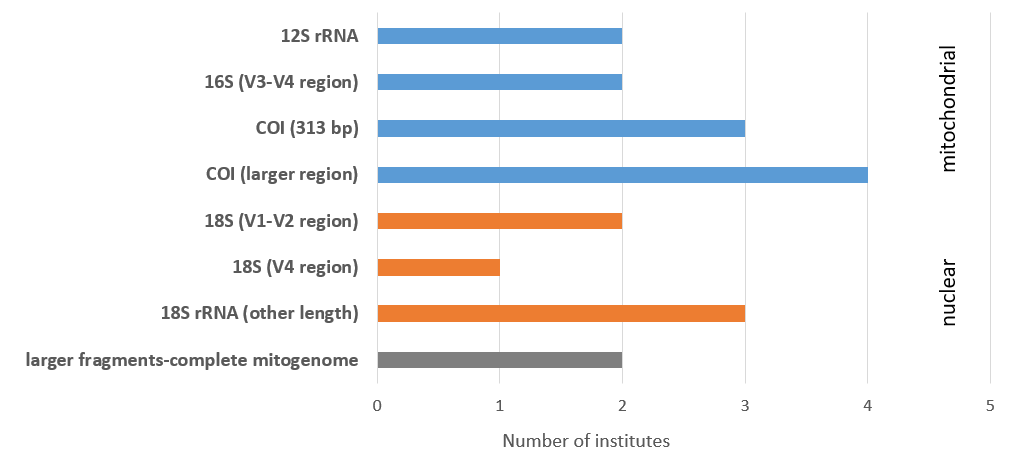


**Figure S5.** The DNA markers used as target region by the GEANS institutes and the number of institutes doing so.


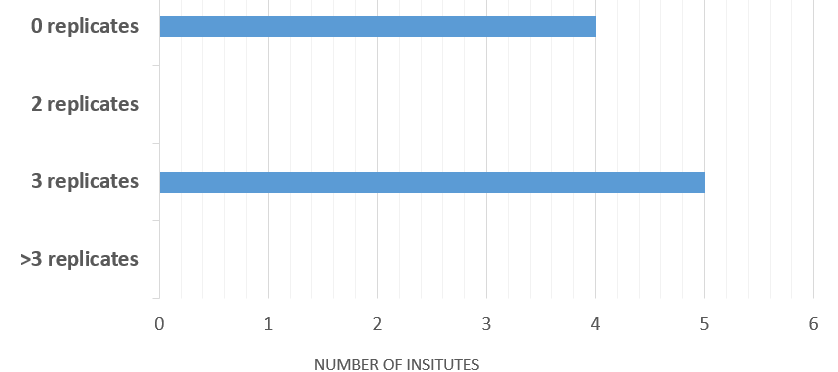


**Figure S6.** The use of PCR replicates by the GEANS institutes.

**Table S1.** The sequencing platforms used by the GEANS institutes.

| **Institute** | **What platform do you use for amplicon sequencing?** | **What are your reasons for using this method?** |
| --- | --- | --- |
| Institute 1 | Illumina sequencing, Nanopore sequencing | Standard method at institute initially Illumina MiSeq or NextSeq depending on sequence length, now also introducing Nanopore but mostly for very long reads |
| Institute 2 | Illumina sequencing | Standard method at institute (the machine that we have available) |
| Institute 3 | Illumina sequencing | Well established method, cost efficient |
| Institute 4 | Illumina sequencing | Standard, costs, collaboration sequencing company |
| Institute 5 | Illumina sequencing, Ion Torrent sequencing | Standard method is Illumina, but Ion Torrent can be better for amplicon sequencing, if amplicons only are to be sequenced |
| Institute 6 | Illumina sequencing | Precision |
| Institute 7 | Illumina sequencing, Nanopore sequencing | Illumina is cost effective and has well established protocols. Nanopore is disappointing |
| Institute 8 | Illumina sequencing | Standard method |
| Institute 9 | Nanopore sequencing | Costs, time to results, portability, read length |
